# Serum Metabolomic Profiling in Rheumatoid Arthritis Patients With Interstitial Lung Disease: A Case–Control Study

**DOI:** 10.3389/fmed.2020.599794

**Published:** 2020-12-17

**Authors:** Hiroshi Furukawa, Shomi Oka, Kota Shimada, Akira Okamoto, Atsushi Hashimoto, Akiko Komiya, Koichiro Saisho, Norie Yoshikawa, Masao Katayama, Toshihiro Matsui, Naoshi Fukui, Kiyoshi Migita, Shigeto Tohma

**Affiliations:** ^1^Department of Rheumatology, National Hospital Organization Tokyo National Hospital, Kiyose, Japan; ^2^Clinical Research Center for Allergy and Rheumatology, National Hospital Organization Sagamihara National Hospital, Sagamihara, Japan; ^3^Department of Rheumatology, National Hospital Organization Sagamihara National Hospital, Sagamihara, Japan; ^4^Department of Rheumatic Diseases, Tokyo Metropolitan Tama Medical Center, Fuchu, Japan; ^5^Department of Rheumatology, National Hospital Organization Himeji Medical Center, Himeji, Japan; ^6^Department of Internal Medicine, Sagami Seikyou Hospital, Sagamihara, Japan; ^7^Department of Clinical Laboratory, National Hospital Organization Sagamihara National Hospital, Sagamihara, Japan; ^8^Department of Orthopedics/Rheumatology, National Hospital Organization Miyakonojo Medical Center, Miyakonojo, Japan; ^9^Tanimura Hospital, Nobeoka, Japan; ^10^Department of Internal Medicine, National Hospital Organization Nagoya Medical Center, Nagoya, Japan; ^11^Clinical Research Center, National Hospital Organization Nagasaki Medical Center, Ōmura, Japan; ^12^Department of Gastroenterology and Rheumatology, Fukushima Medical University School of Medicine, Fukushima, Japan

**Keywords:** rheumatoid arthritis, interstitial lung disease, metabolomics, usual interstitial pneumonia, non-specific interstitial pneumonia

## Abstract

**Objectives:** Interstitial lung disease (ILD) is an extra-articular manifestation in rheumatoid arthritis (RA), detected in 10.7% of patients, and causing a poor prognosis. Hence, biomarkers for ILD are urgently required in RA. Low molecular weight metabolites can be assessed by metabolomic analyses, and although these have been conducted in RA and in idiopathic pulmonary fibrosis, few have been carried out for ILD in the context of RA. Therefore, we analyzed serum metabolomic profiles of ILD in RA to identify novel biomarkers.

**Methods:** Serum samples from 100 RA patients with ILD and 100 matched RA patients without chronic lung disease (CLD) were collected. These samples were subjected to metabolomic analyses using capillary electrophoresis time-of-flight mass spectrometry.

**Results:** A total of 299 metabolites were detected in the metabolomic analysis. By univariate analysis, serum levels of decanoic acid and morpholine were lower in RA with ILD (false discovery rate *Q* = 1.87 × 10^−11^ and 7.09 × 10^−6^, respectively), and glycerol was higher (*Q* = 1.20 × 10^−6^), relative to RA without CLD. Serum levels of these metabolites in RA with usual interstitial pneumonia or RA with non-specific interstitial pneumonia were also altered. The partial least squares-discriminant analysis model generated from these three metabolites could successfully discriminate ILD in RA (area under the curve: 0.919, 95% confidence interval: 0.867–0.968, sensitivity 0.880, specificity 0.780).

**Conclusions:** Serum levels of some metabolites were significantly different in RA with ILD compared with RA without CLD. It is concluded that metabolomic profiling will be useful for discovering candidate screening biomarkers for ILD in RA.

## Introduction

Rheumatoid arthritis (RA) is a systemic autoimmune disease affecting synovial joints, but extra-articular manifestations are also observed. These include interstitial lung disease (ILD), characterized by interstitial inflammation of the lung, in 10.7% of patients (1). ILD predicts a poor prognosis in RA (2). Krebs von den lungen-6 (KL-6) and surfactant protein-D (SP-D) have been used as biomarkers for ILD. However, their cutoff levels were higher for their application to RA-associated ILD (RA-ILD), and their sensitivity is insufficient (3). Many studies of ILD markers have been reported in idiopathic pulmonary fibrosis, but few have been validated for RA-ILD. Thus, biomarkers for ILD in RA patients are needed.

Low molecular weight metabolites are commonly analyzed to elucidate altered metabolisms in pathological conditions. Systematic investigations of metabolic pathways and low molecular weight compounds have mostly been conducted to clarify the pathogenesis of cancer and to generate cancer biomarkers (4). Some metabolomic analyses were also conducted separately in RA (5) or idiopathic pulmonary fibrosis (6–8) but rarely for RA-ILD. Here, we analyzed serum metabolomic profiles of ILD in RA to generate novel biomarkers for RA-ILD.

## Methods

### Patients

A total of 200 RA patients with chest computed tomography (CT) images were recruited at Himeji Medical Center, Miyakonojo Medical Center, Nagasaki Medical Center, Nagoya Medical Center, and Sagamihara National Hospital. All patients fulfilled the 2010 American College of Rheumatology/European League Against Rheumatism classification criteria (9) or 1987 American College of Rheumatology classification criteria for RA (10). RA patients were diagnosed with usual interstitial pneumonia (UIP); irregular linear opacities and honeycombing, non-specific interstitial pneumonia (NSIP); bilateral ground-glass attenuation patterns predominantly in subpleural and basal regions or no chronic lung diseases (CLDs); and no abnormalities in CT images, as previously described (11). RA patients with UIP [UIP(+)RA, case 1, cases 101–146] or NSIP [NSIP(+)RA, case 2, cases 201–254] were designated as RA with ILD [ILD(+)RA, case 1 + 2, cases 101–146, cases 201–254], and each control RA patient without CLD [CLD(–)RA, control 1 + 2, controls 101–146, controls 201–254] was matched for age group (30–39, 40–49, 50–59, 60–69, 70–79, 80–89), sex, and the use of corticosteroids, conventional synthetic disease-modifying antirheumatic drugs (csDMARDs), or biological/targeted synthetic disease-modifying antirheumatic drugs (b/tsDMARDs) in this multicenter retrospective case–control observational study. CLD(–)RA matched for UIP(+)RA (case 1) or NSIP(+)RA (case 2) was designated as control 1 or control 2, respectively. Serum samples were collected from these cases and control RA patients. Rheumatoid factor was detected by N-latex RF kit (Siemens Healthcare Diagnostics, München, Germany), and anti-citrullinated peptide antibody was measured by Mesacup-2 test CCP (Medical & Biological Laboratories, Nagoya, Japan). KL-6 and SP-D were measured using a Picolumi KL-6 Electrochemiluminescence immunoassay system (EIDIA Co., Ltd., Tokyo, Japan) and the SP-D kit “Yamasa” EIA II (Yamasa Corporation, Choshi, Japan), respectively. This study was reviewed and approved by the NHO Central Institutional Review Board. Written informed consent was obtained from all study participants. This study was conducted in accordance with the principles expressed in the Declaration of Helsinki.

### Serum Metabolomic Profiles

Serum metabolomic profiles were analyzed with capillary electrophoresis time-of-flight mass spectrometry using an Agilent CE capillary electrophoresis system equipped with an Agilent 6210 time-of-flight mass spectrometer, Agilent 1100 isocratic HPLC pump, Agilent G1603A CE-MS adapter kit, and Agilent G1607A CE-ESI-MS sprayer kit (Agilent Technologies, Waldbronn, Germany) at Human Metabolome Technologies (Tsuruoka, Japan) (12–14). Serum metabolomic analyses were performed by MetaboAnalyst 4.0 (https://www.metaboanalyst.ca/MetaboAnalyst/home.xhtml) (15). Zero values were replaced with half of the minimum positive value. Auto scaling was conducted as mean-centered and divided by the standard deviation of each metabolite level. Hierarchical cluster analysis was performed by Ward's method and Euclidean distance, and heat maps with dendrograms were generated by Bell Curve for Excel software (Social Survey Research Information Co., Ltd., Tokyo, Japan). Univariate analyses were performed, and Student's *t*-test was conducted for the comparison of normalized metabolite levels. Multiple testing was corrected by calculating false discovery rate (FDR) *Q* values. Receiver operator characteristic (ROC) curves were developed, and area under the curve (AUC) values were calculated. Partial least squares-discriminant analysis (PLS-DA) was performed to select candidate metabolites from variable importance in projection (VIP) scores. Multivariate analysis was conducted with PLS-DA to exploit compound metabolite biomarkers for RA-ILD from three selected metabolites, and Monte Carlo cross-validation was performed. The impact of ILD in RA on metabolic pathways was validated with *Homo sapiens* pathway library, global test, and relative-betweenness centrality, as previously analyzed (14). Pathway enrichment analysis was performed to identify concentration changes of metabolites involved in the same biological pathway. The pathway topology analysis was conducted to estimate node importance indicating more important positions of a biological pathway network of metabolites. The *P-*values from the pathway enrichment analysis and the pathway impact values from the pathway topology analysis were calculated by MetaboAnalyst 4.0 and plotted as pathway analysis results.

## Results

### Characteristics of the RA Patients

The levels of rheumatoid factor, KL-6, and SP-D in ILD(+)RA were higher than those in CLD(–)RA (Table 1), although CLD(–) RA was matched for age group, sex, and the use of corticosteroids, csDMARDs, or b/tsDMARDs. There were no significant differences in terms of age at onset, Steinbrocker stage, ever having smoked, or anti-citrullinated peptide antibody levels between ILD(+)RA and CLD(–)RA. The levels of rheumatoid factor, KL-6, and SP-D in UIP(+)RA or NSIP(+)RA were also higher than those in matched controls of CLD(–)RA (Table 1).

**Table 1:** Characteristics of RA patients.

	**ILD(+) RA** ** case 1 + 2**	**CLD(–)RA control 1 + 2**	***P***	**UIP(+)RA** ** case 1**	**CLD(–)RA control 1**	***P***	**NSIP(+)RA** ** case 2**	**CLD(–)RA control 2**	***P***
Number	100	100		46	46		54	54	
Mean age, years (SD)	67.3 (8.5)	66.2 (8.9)	0.3678	67.2 (9.1)	66.6 (9.0)	0.7473	67.3 (8.1)	65.8 (8.8)	0.3481
Male, *n* (%)	24 (24.0)	24 (24.0)	1.0000^*^	16 (34.8)	16 (34.8)	1.0000^*^	8 (14.8)	8 (14.8)	1.0000^*^
Age at onset, years (SD)	54.0 (13.6)	51.7 (12.7)	0.2205	54.0 (14.3)	51.7 (13.6)	0.4349	54.1 (13.0)	51.8 (12.1)	0.3456
Steinbrocker stage III and IV, *n* (%)	49 (49.0)	54 (54.5)	0.4792^*^	25 (54.3)	24 (52.2)	1.0000^*^	24 (44.4)	30 (56.6)	0.2480^*^
Smoker or past smoker, *n* (%)	37 (38.1)	29 (32.6)	0.4471^*^	18 (40.9)	18 (45.0)	0.8258^*^	19 (35.8)	11 (22.4)	0.1919^*^
RF, IU/ml (SD)	564.6 (1,302.2)	154.3 (241.2)	0.0022	523.0 (1,009.2)	138.0 (205.6)	0.0131	598.5 (1,508.2)	168.1 (269.0)	0.0414
ACPA, IU/ml (SD)	374.6 (830.3)	252.5 (293.4)	0.1680	289.0 (302.6)	269.5 (290.5)	0.7574	442.7 (1,079.5)	238.0 (297.8)	0.1821
KL-6, U/ml (SD)	762.9 (647.5)	322.7 (400.4)	1.41 × 10^−5^	749.9 (652.6)	277.5 (128.1)	0.0014	772.9 (649.5)	354.9 (513.7)	0.0029
SP-D, ng/ml (SD)	128.8 (148.3)	46.1 (31.3)	0.0002	128.6 (92.6)	48.6 (23.1)	0.0004	129.0 (182.7)	44.2 (36.3)	0.0179
Corticosteroid administration, *n* (%)	75 (75.0)	75 (75.0)	1.0000^*^	36 (78.3)	36 (78.3)	1.0000^*^	39 (72.2)	39 (72.2)	1.0000^*^
csDMARDs administration, *n* (%)	86 (86.0)	86 (86.0)	1.0000^*^	42 (91.3)	42 (91.3)	1.0000^*^	44 (81.5)	44 (81.5)	1.0000^*^
b/tsDMARDs administration, *n* (%)	27 (27.0)	27 (27.0)	1.0000^*^	13 (28.3)	13 (28.3)	1.0000^*^	14 (25.9)	14 (25.9)	1.0000^*^

RA, rheumatoid arthritis; ILD, interstitial lung disease; UIP, usual interstitial pneumonia; NSIP, non-specific interstitial pneumonia; CLD, chronic lung disease; SD, standard deviation; RF, Rheumatoid factor; ACPA, Anti-citrullinated peptide antibody; KL-6, Krebs von den lungen-6; SP-D, Surfactant protein-D; csDMARDs, conventional synthetic disease-modifying antirheumatic drugs; b/tsDMARDs, biological/targeted synthetic disease-modifying antirheumatic drugs; ILD(+)RA, RA patients with ILD; CLD(–)RA, RA patients without CLD; UIP(+)RA, RA patients with UIP; NSIP(+)RA, RA patients with NSIP. Numbers or average values of each group are shown. Standard deviations or percentages are shown in parenthesis. Difference was tested in the comparison with the CLD(–) population by Fisher's exact test using 2 × 2 contingency tables or Student's t-test.

* *Fisher's exact test was employed*.

### Serum Metabolomic Profiles of RA-ILD

A total of 299 metabolites were detected in the metabolomic analysis (Supplementary Table 1). Two hundred five metabolites were compared between ILD(+)RA and CLD(–)RA after normalization with data scaling (Supplementary Table 2). In the univariate analyses, significant differences were detected between the two groups of patients. Serum levels of some metabolites in UIP(+)RA or NSIP(+)RA were also altered (Supplementary Table 2). Meta-analysis of these comparisons was performed, and significant differences were still present after multiple testing corrected by FDR (Table 2). The levels of decanoic acid (FDR *Q* = 1.87 × 10^−11^) and morpholine (FDR *Q* = 7.09 × 10^−6^) were lower in ILD(+)RA, and the levels of glycerol were higher (FDR *Q* = 1.20 × 10^−6^) than in CLD(–)RA. These metabolites were clustered and visualized in a heat map (Supplementary Figure 1). Hierarchical cluster analysis of the metabolites did not indicate any apparent discrimination of ILD(+)RA. Thus, serum levels of some metabolites were significantly skewed in ILD(+)RA, although no apparent change was observed in serum levels of most metabolites.

**Table 2:** Metabolites associated with ILD in RA.

	**ILD(+)RA case 1 + 2**	**CLD(–)RA control 1 + 2**	**Case 1 + 2 vs. control 1 + 2**	**UIP(+)RA case 1**	**CLD(–)RA control 1**	**Case 1 vs. control 1**	**NSIP(+)RA case 2**	**CLD(–)RA control 2**	**Case 2 vs. control 2**	**Meta-analysis of (case 1 vs. control 1) and (case 2 vs. control 2)**	**Meta-analysis of (case 1 vs. control 1) and (case 2 vs. control 2)**
			***P***			***P***			***P***	***P***	**FDR *Q***
Decanoic acid	−0.58 (0.62)	0.59 (0.97)	3.85 × 10^−19^	−0.57 (0.63)	0.66 (1.06)	3.92 × 10^−9^	−0.58 (0.62)	0.53 (0.90)	2.07 × 10^−11^	9.10 × 10^−14^	1.87 × 10^−11^
Glycerol	0.44 (0.99)	−0.43 (0.78)	3.77 × 10^−11^	0.52 (0.89)	−0.60 (0.75)	2.17 × 10^−9^	0.38 (1.07)	−0.29 (0.79)	0.0004	1.17 × 10^−8^	1.20 × 10^−6^
Morpholine	−0.43 (0.00)	0.44 (1.28)	1.97 × 10^−10^	−0.43 (0.00)	0.33 (1.21)	7.37 × 10^−5^	−0.43 (0.00)	0.53 (1.34)	7.44 × 10^−7^	1.04 × 10^−7^	7.09 × 10^−6^
Dyphylline	0.37 (0.99)	−0.36 (0.87)	6.45 × 10^−8^	0.42 (0.93)	−0.60 (0.82)	1.44 × 10^−7^	0.33 (1.04)	−0.16 (0.87)	0.0099	2.04 × 10^−6^	0.0001
Octanoic acid	−0.32 (0.91)	0.33 (0.99)	2.78 × 10^−6^	−0.27 (0.94)	0.55 (1.12)	0.0003	−0.37 (0.89)	0.14 (0.83)	0.0027	0.0002	0.0074
Fumaric acid monomethyl ester	−0.30 (0.81)	0.31 (1.08)	2.01 × 10^−5^	−0.35 (0.80)	0.43 (1.00)	0.0002	−0.25 (0.82)	0.21 (1.14)	0.0187	0.0006	0.0192
N-Acetylgalactosamine-1 N-Acetylmannosamine-1 N-Acetylglucosamine-1	0.29 (1.04)	−0.29 (0.87)	2.46 × 10^−5^	0.18 (0.94)	−0.41 (0.77)	0.0015	0.39 (1.12)	−0.19 (0.94)	0.0040	0.0008	0.0226

*Average values of the normalized data of each group are shown. Standard deviations are shown in parenthesis. Association was tested between cases and controls by Student's t-test. Meta-analysis of UIP and NSIP was performed. To correct for multiple testing; the false discovery rate (FDR) Q value was calculated. ILD, interstitial lung disease; CLD, chronic lung disease; RA, rheumatoid arthritis; UIP, usual interstitial pneumonia; NSIP, non-specific interstitial pneumonia; ILD(+)RA, RA patients with ILD; CLD(–)RA, RA patients without CLD; UIP(+)RA, RA patients with UIP; NSIP(+)RA, RA patients with NSIP; FDR, false discovery rate*.

### Potential Biomarkers for RA-ILD

**Figure 1:**
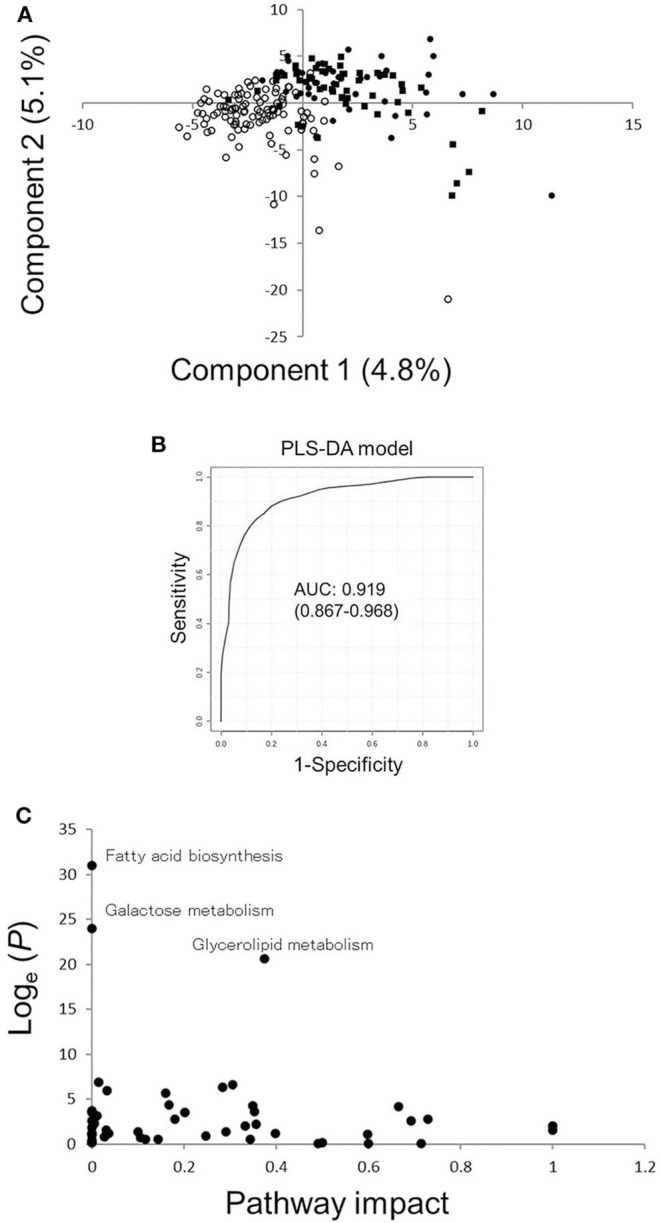
Results of PLS-DA and pathway analyses. **(A)** Score plots for PLS-DA analysis in RA patients with ILD or without CLD. Filled circles, filled squares, and open circles indicate the results of sera from RA patients with UIP or NSIP or without CLD, respectively. **(B)** ROC curves of the PLS-DA model with three metabolites with higher VIP scores comparing RA patients with ILD or without CLD. The AUC value of the ROC curve is 0.919, and the 95% confidence interval of the AUC is 0.867–0.968. **(C)** Pathway analysis based on metabolites in sera from RA patients with ILD or without CLD. A *P*-value from pathway enrichment analysis and a pathway impact value from pathway topology analysis were plotted. PLS-DA, partial least squares-discriminant analysis; RA, rheumatoid arthritis; ILD, interstitial lung disease; CLD, chronic lung disease; ILD(+)RA, RA patients with ILD; CLD(–)RA, RA patients without CLD; AUC, area under the curve; ROC, receiver operating characteristic; VIP, variable importance in projection.

PLS-DA was conducted to generate potential biomarkers for ILD in RA (Figure 1A). ILD in RA was successfully discriminated by component 1, for which VIP scores were calculated (Supplementary Table 2). Three metabolites with VIP scores >3.5 (decanoic acid, glycerol, morpholine) were selected to develop new biomarkers for ILD in RA. ROC curves of these three metabolites were generated, and AUC values were calculated [Supplementary Figure 2, decanoic acid: AUC 0.800, 95% confidence interval [CI] 0.747–0.858, glycerol: AUC 0.772, 95% CI 0.711–0.829, morpholine: AUC 0.660, 95% CI 0.615–0.705]. A PLS-DA model was created with these three metabolites, and a ROC curve of the PLS-DA was generated with an AUC of 0.919 (95% CI 0.867–0.968) (Figure 1B). The average accuracy based on 100 cross-validations was 0.838, and a permutation test was carried out (Permutation *P* = 1.72 × 10^−5^). The optimized condition estimated from the ROC curve provided a sensitivity of 0.880 and specificity of 0.780 (Supplementary Figure 3). Since the prevalence of ILD in RA in our previous study was 0.107 (1), positive and negative predictive values of the created biomarker model were calculated to be 0.331 and 0.981, respectively. The impact of ILD in RA on metabolic pathways was validated, and glycerolipid metabolism, fatty acid biosynthesis, and galactose metabolism were shown to be influenced by ILD in RA (Figure 1C). Thus, the PLS-DA model with the three metabolites generated the candidate screening biomarker with high sensitivity, and it can be concluded that some metabolic pathways were influenced by ILD in RA.

## Discussion

The development of a single biomarker for the diagnosis of multifactorial diseases would be difficult, although metabolomic biomarkers have been explored in this context over the years. In the present study, serum levels of some metabolites including decanoic acid, glycerol, and morpholine were found to be different in RA patients with or without ILD. Different levels of three metabolites in the serum were confirmed in both UIP-associated RA and NSIP-associated RA. PLS-DA was also used for the generation of robust complex markers of RA-ILD with these three metabolites compared with current ILD markers. Altered lipid metabolism has been explored in the pathogenesis of RA-ILD, as was also reported in idiopathic pulmonary fibrosis (6, 8). Plasma decanoic acid and octanoic acid levels were reported to increase after Roux-en-Y gastric bypass surgery in insulin-resistant patients (16). Because diabetes mellitus is a risk factor for idiopathic pulmonary fibrosis (17), our results could be explained by altered carbohydrate metabolism in the pathogenesis of ILD. Additionally, serum decanoic acid levels were decreased, and glycerol levels were increased in rats gavaged with paraquat, which causes ILD (18), suggesting common mechanisms in RA-ILD and paraquat-induced ILD. Decanoic acid and octanoic acid levels have also been reported to be increased in colorectal cancer patient sera and breast cancer tissues (19, 20). Serum decanoic acid levels were increased in lupus nephritis as well (21), suggesting the presence of contrasting metabolic conditions in cancer or inflammation compared with RA-ILD. Although altered levels of glycerol have been reported in metabolomic analyses in several diseases (22), few studies have noted altered morpholine levels. The different profiles of these metabolites in RA with or without ILD would reflect the altered glycerolipid metabolism in ILD. However, it is difficult to investigate causality, because no animal model for RA-ILD has been established.

Lung metabolomic analyses were performed in idiopathic pulmonary fibrosis patients (6–8). An apparent difference of metabolomic profiles was observed in these studies. Serum metabolomic analyses were also conducted in RA (5), and obviously, different metabolomic profiles were reported. Profiles of some metabolites were significantly different between ILD(+)RA and CLD(–)RA, but the number of the metabolites with significant differences between ILD(+)RA and CLD(–)RA seemed to be smaller than the difference between idiopathic pulmonary fibrosis patients and healthy controls or RA and healthy controls. The comparison with healthy controls might reveal relatively larger differences, and the comparison between disease subsets could only show the essential differences.

Although the sample size of the present study is modest, the results obtained from UIP-associated RA were replicated in NSIP-associated RA. Nevertheless, independent larger scale studies are necessary to replicate the results obtained from the present preliminary study. Metabolomic profiles in other potential controls, such as idiopathic pulmonary fibrosis patients, RA patients with emphysema or airway diseases, or healthy controls, were not compared with RA-ILD in the present study. In future studies, these comparisons should be performed to discriminate RA-ILD from other conditions. Although cases and controls were matched for age, sex, and the use of corticosteroids and disease-modifying antirheumatic drugs in this study, the serum metabolite levels might be influenced by other unmatched factors including disease duration of ILD, comorbidities, disease activity score in 28 joints, health assessment questionnaire disability index, or drug dosage or duration. The effect sizes of these factors would be clarified in future studies. Nonetheless, to the best of our knowledge, this is the first metabolomic analysis to discriminate RA-ILD from RA without lung disease. Thus, the results of univariate analyses showed significantly different serum levels of some metabolites (decanoic acid, glycerol, morpholine) in RA patients with ILD relative to those without lung disease. Additionally, PLS-DA analyses provided candidate screening biomarkers for RA-ILD generated from these three metabolites and offer better sensitivity (0.880) than current biomarkers for ILD. Metabolomic profiling would be useful to generate better biomarkers for ILD in RA.

## Data Availability Statement

The original contributions presented in the study are included in the article/Supplementary Material, further inquiries can be directed to the corresponding author/s.

## Ethics Statement

The studies involving human participants were reviewed and approved by the NHO Central Institutional Review Board. The patients/participants provided their written informed consent to participate in this study.

## Author Contributions

HF and ST conceived, designed the experiments, and contributed to the writing of the manuscript. HF and SO performed the experiments. HF analyzed the data. HF, KSh, AO, AH, AK, KSa, NY, MK, TM, NF, KM, and ST contributed reagents/materials/analysis tools. All authors read and approved the final manuscript.

## Conflict of Interest

HF was supported by research grants from the following funders supported wholly or in part by the indicated pharmaceutical companies: The Japan Research Foundation for Clinical Pharmacology, run by Daiichi Sankyo; the Takeda Science Foundation supported by an endowment from Takeda Pharmaceutical Company; and the Nakatomi Foundation, established by Hisamitsu Pharmaceutical Co., Inc. The Daiwa Securities Health Foundation was established by Daiwa Securities Group Inc. and Mitsui Sumitomo Insurance Welfare Foundation was established by Mitsui Sumitomo Insurance Co., Ltd. HF was supported by research grants from Bristol-Myers-Squibb Co. HF received honoraria from Ajinomoto Co., Inc., Daiichi Sankyo Co., Ltd., Dainippon Sumitomo Pharma Co., Ltd., Pfizer Japan Inc., and Takeda Pharmaceutical Company, Luminex Japan Corporation Ltd., and Ayumi Pharmaceutical Corporation. ST was supported by research grants from nine pharmaceutical companies: Abbott Japan Co., Ltd., Astellas Pharma Inc., Chugai Pharmaceutical Co., Ltd., Eisai Co., Ltd., Mitsubishi Tanabe Pharma Corporation, Merck Sharp and Dohme Inc., Pfizer Japan Inc., Takeda Pharmaceutical Company Limited, Teijin Pharma Limited. ST received honoraria from Asahi Kasei Pharma Corporation, Astellas Pharma Inc., AbbVie GK., Chugai Pharmaceutical Co., Ltd., Ono Pharmaceutical Co., Ltd., Mitsubishi Tanabe Pharma Corporation, Pfizer Japan Inc. The remaining authors declare that the research was conducted in the absence of any commercial or financial relationships that could be construed as a potential conflict of interest.
