# Supplementary material for: Serum Metabolomic Profiling in Rheumatoid Arthritis Patients With Interstitial Lung Disease: A Case–Control Study
Source: Front Med (Lausanne). 2020 Dec 17;7:599794. doi: 10.3389/fmed.2020.599794 (PMC7773768; doi:10.3389/fmed.2020.599794)
Supplement: Supplementary file 1 [file Table_1.pdf]

Supplemental Table S1. Metabolomic profile of detected metabolites

		Case									
ID	Compound name	case 101	case 102	case 103	case 104	case 105	case 106	case 107	case 108	case 109	[illegible]
C_0058	1-Aminocyclopentanecarboxylic acid	ND	ND	ND	ND	ND	ND	ND	ND	ND	[illegible]
C_0019	1-Methyl-2-pyrrolidone	ND	ND	ND	ND	ND	ND	ND	ND	ND	[illegible]
C_0085	1-Methyl-4-imidazoleacetic acid	0.000134	8.2E-05	0.000216	0.000127	0.000128	0.000122	0.000125	0.000122	0.000152	[illegible]
C_0192	1-Methyladenosine	6.77E-05	5.47E-05	9.36E-05	5.17E-05	0.000122	6.52E-05	5.72E-05	6.83E-05	8.77E-05	[illegible]
C_0120	1-Methylhistidine3-Methylhistidine	0.004812	0.001642	0.004126	0.00295	0.003163	0.001228	0.003347	0.002047	0.002167	[illegible]
C_0078	1-Methylnicotinamide	3.25E-05	3.41E-05	2.35E-05	6.27E-05	4.44E-05	4.63E-05	0.000107	4.59E-05	ND	[illegible]
C_0034	1-Pyrroline 5-carboxylic acid	2.17E-05	3.18E-05	ND	ND	ND	ND	ND	ND	ND	[illegible]
C_0084	1H-Imidazole-4-propionic acid	ND	ND	ND	ND	4.13E-05	ND	2.91E-05	ND	ND	[illegible]
C_0169	2'-Deoxyuridine	ND	ND	ND	ND	ND	ND	ND	ND	ND	[illegible]
C_0018	2,5-Pyrroledione	0.000549	0.000409	0.000375	0.000326	0.00035	0.000364	0.000362	0.000491	0.000464	[illegible]
C_0150	2,6-Diaminopimelic acid	ND	ND	ND	2.29E-05	ND	ND	ND	ND	ND	[illegible]
C_0047	2-Amino-2-(hydroxymethyl)-1,3-propanediol	ND	ND	ND	ND	ND	ND	ND	ND	ND	[illegible]
C_0025	2-Aminoisobutyric acid2-Aminobutyric acid	0.003274	0.00628	0.003287	0.010341	0.006216	0.007189	0.00338	0.002796	0.004977	[illegible]
A_0016	2-Furoic acid	ND	ND	ND	ND	ND	ND	ND	ND	ND	[illegible]
A_0037	2-Hydroxy-4-methylvaleric acid	ND	ND	0.000143	ND	ND	ND	0.000107	ND	ND	[illegible]
A_0013	2-Hydroxybutyric acid	0.002489	0.002648	0.007906	0.0071	0.008297	0.00273	0.001685	0.002068	0.002597	[illegible]
A_0047	2-Hydroxyglutaric acid	ND	ND	ND	ND	0.000152	ND	ND	ND	0.000149	[illegible]
A_0011	2-Hydroxyisobutyric acid	0.000423	ND	0.000511	0.000443	0.00059	ND	ND	0.000261	0.000414	[illegible]
A_0054	2-Hydroxyoctanoic acid-2	ND	ND	ND	ND	ND	ND	ND	ND	ND	[illegible]
A_0024	2-Hydroxyvaleric acid	0.001467	0.000789	0.001444	0.000942	0.001583	0.00098	0.000619	0.001368	0.000782	[illegible]
A_0073	2-Isopropylmalic acid	ND	ND	ND	ND	ND	ND	ND	ND	ND	[illegible]
C_0041	2-Methylserine	ND	ND	ND	ND	ND	ND	ND	ND	7.92E-05	[illegible]
A_0046	2-Oxoglutaric acid	0.000787	0.000918	0.001052	0.00132	0.001131	0.000974	ND	ND	ND	[illegible]
A_0019	2-Oxoisovaleric acid	0.001585	0.001704	0.002191	0.001884	0.002065	0.001804	0.001066	0.001386	0.001351	[illegible]
A_0052	2-Oxooctanoic acid	ND	ND	ND	ND	ND	ND	ND	ND	ND	[illegible]
C_0036	3-Amino-2-piperidone	0.000104	6.38E-05	0.000125	7.03E-05	0.000112	4.8E-05	8.53E-05	8.09E-05	7.65E-05	[illegible]
C_0024	3-Aminoisobutyric acid	0.000248	0.000593	0.001902	0.000236	0.000509	0.000561	0.001032	0.001528	0.000548	[illegible]
A_0012	3-Hydroxybutyric acid	0.001389	0.00175	0.033207	0.007053	0.003547	0.001487	0.001126	0.001363	0.002719	[illegible]
A_0007	3-Hydroxypropionic acid	ND	ND	ND	ND	ND	ND	ND	ND	ND	[illegible]
A_0097	3-Indoxylsulfuric acid	0.000554	0.000879	0.001453	0.000865	ND	0.001222	0.00101	0.001268	0.000885	[illegible]
C_0161	3-Methoxytyrosine	ND	3.17E-05	ND	3.74E-05	3.76E-05	ND	ND	ND	4.14E-05	[illegible]
A_0035	3-Ureidopropionic acid	ND	ND	ND	ND	ND	ND	ND	ND	ND	[illegible]
C_0103	4-(β-Acetylaminoethyl)imidazole	ND	ND	ND	ND	ND	ND	4.32E-05	ND	ND	[illegible]
A_0045	4-Acetamidobutanoic acid	ND	ND	0.000106	ND	ND	ND	ND	ND	ND	[illegible]
C_0151	4-Aminohippuric acid	ND	ND	ND	ND	ND	ND	ND	ND	ND	[illegible]
C_0089	4-Guanidinobutyric acid	ND	4.7E-05	ND	ND	ND	ND	ND	ND	ND	[illegible]
A_0031	4-Methyl-2-oxovaleric acid3-Methyl-2-oxovaleric acid	0.00416	0.007033	0.007647	0.009125	0.009046	0.006494	0.004583	0.00522	0.006853	[illegible]
A_0018	4-Oxovaleric acid	ND	ND	ND	ND	ND	ND	0.000222	ND	ND	[illegible]
A_0077	4-Pyridoxic acid	ND	ND	ND	8.75E-05	ND	ND	ND	ND	ND	[illegible]
C_0065	5-Amino-4-oxovaleric acid	ND	ND	ND	ND	4.4E-05	ND	ND	ND	ND	[illegible]
C_0071	5-Aminoindole	ND	ND	ND	ND	ND	ND	ND	ND	ND	[illegible]
C_0111	5-Hydroxylysine	0.000116	0.000111	5.13E-05	7.26E-05	0.000109	8.6E-05	8.71E-05	0.000103	0.000104	[illegible]
A_0093	5-Methoxyindoleacetic acid	ND	ND	0.0002	ND	0.000157	ND	ND	ND	0.000188	[illegible]
A_0029	5-Oxo-2-tetrahydrofurancarboxylic acid	ND	ND	ND	ND	ND	0.000185	ND	ND	ND	[illegible]
A_0027	5-Oxoproline	0.002454	0.003009	0.003191	0.002841	0.002099	0.002862	0.002374	0.002758	0.001599	[illegible]
A_0036	6-Hydroxyhexanoic acid	ND	ND	0.00029	ND	ND	ND	ND	ND	ND	[illegible]
C_0115	7-Methylguanine	5.66E-05	2.61E-05	5.1E-05	ND	4.1E-05	ND	ND	3.03E-05	4.07E-05	[illegible]
C_0194	8-Hydroxy-2'-deoxyguanosine	6.62E-05	0.000104	0.000126	0.000136	9.05E-05	9E-05	7.84E-05	0.000108	0.000112	[illegible]
A_0055	8-Hydroxyoctanoic acid2-Hydroxyoctanoic acid-1	ND	ND	0.00016	ND	ND	ND	ND	ND	ND	[illegible]
C_0021	Acetoacetamide	ND	ND	ND	ND	ND	ND	ND	ND	ND	[illegible]
A_0009	Acetoacetic acid	ND	ND	0.000434	ND	ND	ND	ND	ND	0.000308	[illegible]
C_0006	Acetohydrazide	0.000923	0.00154	0.00387	0.000872	0.002249	ND	7.77E-05	ND	ND	[illegible]
C_0188	Adenosine	ND	ND	ND	ND	ND	ND	ND	ND	8.57E-05	[illegible]
C_0154	ADMA	0.000212	0.000221	0.000153	0.0002	0.000234	0.000265	0.000199	0.000209	0.000222	[illegible]
C_0014	Ala	0.077776	0.099478	0.066258	0.091159	0.087539	0.127546	0.063874	0.06715	0.091344	[illegible]
C_0130	Alliin	0.000254	ND	ND	ND	ND	ND	ND	ND	ND	[illegible]
C_0004	Aminoacetone	0.001101	0.001656	0.001021	0.001382	0.002256	0.001319	0.00111	0.002184	0.001313	[illegible]
C_0138	Aminophylline	ND	ND	0.01255	0.000427	0.000387	0.000391	0.000245	ND	ND	[illegible]
C_0045	Anserine_divalent	ND	ND	ND	ND	ND	ND	ND	ND	ND	[illegible]
C_0079	Anthranilic acid	ND	ND	ND	ND	ND	ND	ND	ND	ND	[illegible]
C_0125	Arg	0.046083	0.047915	0.034215	0.039592	0.047701	0.044716	0.03711	0.047209	0.03545	[illegible]
C_0198	Argininosuccinic acid	2.59E-05	ND	ND	ND	ND	1.63E-05	2.32E-05	ND	ND	[illegible]
A_0105	Ascorbate 2-sulfate	0.000565	0.000452	0.000435	0.001061	0.000448	0.000361	0.000569	0.000298	0.0007	[illegible]
A_0072	Ascorbic acid	ND	ND	ND	ND	ND	ND	ND	ND	ND	[illegible]
C_0069	Asn	0.00758	0.011834	0.007784	0.009565	0.011702	0.01281	0.007381	0.010038	0.008847	[illegible]
C_0074	Asp	0.007522	0.006936	0.007406	0.008711	0.009718	0.006144	0.004497	0.005941	0.001529	[illegible]
A_0081	Azelaic acid	ND	ND	ND	ND	ND	ND	ND	ND	ND	[illegible]
C_0022	Azetidine 2-carboxylic acid	ND	ND	ND	ND	ND	ND	ND	ND	ND	[illegible]
A_0049	Benzoylformic acid	ND	ND	ND	ND	ND	ND	ND	ND	ND	[illegible]
C_0040	Betaine	0.020864	0.02072	0.016538	0.02049	0.020934	0.023107	0.023143	0.024008	0.029846	[illegible]
C_0106	Betonicine	ND	ND	ND	ND	ND	ND	ND	ND	ND	[illegible]
C_0172	Butyrylcarnitine	ND	ND	ND	0.00016	ND	ND	ND	ND	ND	[illegible]
C_0152	Caffeine	0.002686	0.001423	0.000557	0.003721	0.00123	0.003118	0.000945	0.000711	0.001538	[illegible]
C_0128	Canavanine	ND	ND	ND	ND	ND	ND	ND	ND	ND	[illegible]
C_0159	Carboxymethyllysine	ND	ND	ND	4.62E-05	7.92E-05	ND	3.61E-05	4.12E-05	5.32E-05	[illegible]
C_0110	Carnitine	0.022927	0.029556	0.012666	0.026721	0.020542	0.027982	0.024039	0.017648	0.030161	[illegible]
A_0128	Cholic acid	ND	ND	0.000131	0.000417	ND	ND	ND	ND	ND	[illegible]
C_0028	Choline	0.012604	0.012752	0.011143	0.011862	0.011945	0.010596	0.013617	0.017359	0.014947	[illegible]
C_0184	Cimetidine	ND	ND	ND	ND	ND	ND	ND	ND	ND	[illegible]
C_0063	cis-4-Hydroxyproline	0.000121	6.87E-05	0.000103	0.000128	0.000216	9.23E-05	0.000116	0.000152	0.000151	[illegible]
A_0069	cis-Aconitic acid	0.001323	ND	0.002053	0.001428	0.001372	0.001718	0.001716	0.001088	0.001585	[illegible]
A_0030	Citraconic acid	ND	ND	ND	ND	ND	ND	ND	ND	ND	[illegible]
A_0083	Citric acid	0.021421	0.019107	0.028041	0.022198	0.02543	0.024148	0.022492	0.016628	0.034273	[illegible]
C_0127	Citrulline	0.008652	0.007111	0.007596	0.009509	0.014329	0.00661	0.007303	0.008326	0.013125	[illegible]
C_0066	Creatine	0.021051	0.023638	0.005915	0.020955	0.02508	0.016146	0.014279	0.025721	0.018882	[illegible]
C_0035	Creatinine	0.012932	0.01469	0.02292	0.01377	0.028277	0.015847	0.014846	0.011129	0.027172	[illegible]
C_0020	Cyclohexylamine	ND	ND	ND	ND	ND	ND	ND	ND	ND	[illegible]
C_0167	Cystathionine	ND	3.29E-05	0.00018	3.24E-05	0.000159	ND	ND	8.8E-05	8.63E-05	[illegible]
C_0210	Cysteine glutathione disulfide	2.23E-05	ND	ND	ND	ND	ND	ND	ND	0.000109	[illegible]
C_0177	Cystine	0.003408	0.002114	0.003195	0.001751	0.003211	0.001973	0.002369	0.003285	0.012606	[illegible]
C_0032	Cytosine	ND	1.05E-05	ND	ND	ND	ND	1.3E-05	ND	ND	[illegible]
C_0107	DaminozideAla-Ala	ND	ND	ND	ND	ND	ND	ND	ND	ND	[illegible]
A_0067	Decanoic acid	ND	ND	ND	ND	ND	ND	ND	ND	ND	[illegible]
C_0030	Diethanolamine	0.000236	0.000372	0.000548	0.000834	0.000265	0.00053	0.000199	0.000401	0.000436	[illegible]
C_0185	Dyphylline	0.014331	0.011028	0.011024	0.011879	0.010129	0.012054	0.010559	0.012052	0.00963	[illegible]
C_0086	Ectoine	ND	0.000168	4.85E-05	0.000151	ND	ND	4.5E-05	ND	ND	[illegible]
C_0171	Ergothioneine	ND	ND	ND	ND	ND	ND	ND	ND	ND	[illegible]
A_0090	Erythrose 4-phosphate	ND	ND	ND	ND	ND	ND	ND	ND	ND	[illegible]
C_0002	Ethanolamine	0.001186	0.001275	0.001364	0.001291	0.001868	0.001359	0.001274	0.00119	0.001351	[illegible]
A_0042	Ethanolamine phosphate	ND	ND	ND	ND	ND	ND	ND	ND	ND	[illegible]
A_0099	Ethyl glucuronide	ND	ND	ND	ND	ND	ND	ND	ND	ND	[illegible]
A_0057	Formylanthranilic acid	0.000148	ND	ND	ND	ND	ND	ND	0.000168	ND	[illegible]
A_0017	Fumaric acid	ND	ND	ND	ND	ND	ND	ND	ND	ND	[illegible]
A_0028	Fumaric acid monomethyl ester	ND	ND	ND	ND	ND	ND	ND	ND	ND	[illegible]
C_0026	GABA	ND	ND	ND	ND	ND	ND	ND	ND	ND	[illegible]
C_0133	GalactosamineGlucosamine	0.000118	9.72E-05	ND	6.81E-05	7.54E-05	6.22E-05	ND	9.03E-05	0.000105	[illegible]
C_0092	Gln	0.108879	0.113378	0.124918	0.122955	0.107261	0.156834	0.128634	0.151244	0.131943	[illegible]
C_0095	Glu	0.016198	0.018956	0.022717	0.030565	0.042735	0.01632	0.017431	0.012523	0.005682	[illegible]
C_0191	Glu-Glu	3.86E-05	6.1E-05	0.000113	6.09E-05	8.99E-05	4.75E-05	ND	4.69E-05	ND	[illegible]
A_0095	Glucaric acid	ND	ND	ND	ND	0.000245	ND	ND	ND	ND	[illegible]
A_0089	Gluconic acid	0.000494	0.005368	0.000643	0.000516	0.000818	0.00098	0.000489	0.001533	0.000752	[illegible]
C_0131	Gluconolactone	0.000236	0.000229	0.000176	0.000168	0.000338	0.000237	0.000266	ND	0.000347	[illegible]
A_0086	Glucuronic acid-1Galacturonic acid-1	0.000132	ND	0.000284	0.000227	ND	ND	ND	ND	ND	[illegible]
A_0087	Glucuronic acid-2Galacturonic acid-2	0.000289	0.000275	0.00077	0.000287	0.000801	0.000219	0.000287	0.000433	0.000473	[illegible]
A_0034	Glutaric acid	ND	ND	ND	ND	ND	ND	ND	ND	ND	[illegible]
C_0007	Gly	0.039282	0.05145	0.036127	0.040485	0.049955	0.047067	0.04939	0.043819	0.033092	[illegible]
C_0149	Gly-Asp	5.28E-05	ND	5.31E-05	ND	0.000213	ND	ND	ND	ND	[illegible]
C_0070	Gly-Gly	0.000886	0.001009	0.000624	0.00105	0.001068	0.001076	0.000696	0.000691	9.43E-05	[illegible]
A_0014	Glyceric acid	0.001402	0.001829	0.001086	0.003261	0.000763	0.00125	0.002818	0.001216	0.000777	[illegible]
C_0017	Glycerol	0.099935	0.079499	0.095382	0.101599	0.079082	0.10975	0.100569	0.091315	0.056632	[illegible]
A_0066	Glycerol 3-phosphate	ND	ND	0.000146	ND	ND	ND	0.000108	ND	ND	[illegible]
C_0186	Glycerophosphocholine	0.000841	0.000481	0.000543	0.000768	0.000794	0.000876	0.000477	0.001017	0.000487	[illegible]
A_0132	Glycocholic acid	ND	ND	ND	8.6E-05	0.000165	0.000217	ND	ND	0.000126	[illegible]
C_0126	Guanidinosuccinic acid	0.000101	7.01E-05	0.000113	8.43E-05	0.000115	7.39E-05	9.07E-05	7.27E-05	0.000399	[illegible]
C_0038	Guanidoacetic acid	0.000586	0.000586	0.000501	0.000509	0.000575	0.000779	0.000542	0.000442	0.000621	[illegible]
C_0193	Guanosine	0.000124	ND	7.36E-05	4.81E-05	4.76E-05	0.000138	3.6E-05	4.82E-05	0.000437	[illegible]
A_0020	Hexanoic acid	0.000128	ND	0.000103	0.000101	ND	ND	ND	ND	ND	[illegible]
A_0074	Hippuric acid	0.000195	0.000228	0.000133	0.000808	ND	0.001863	0.000138	0.00029	0.000561	[illegible]
C_0104	His	0.01369	0.01914	0.017484	0.019913	0.022829	0.023078	0.019944	0.024397	0.020278	[illegible]
C_0195	His-Glu	ND	ND	ND	ND	1.89E-05	ND	ND	ND	ND	[illegible]
C_0146	Homoarginine	0.000515	0.000551	0.000374	0.000496	0.000597	0.000999	0.000457	0.000489	0.000366	[illegible]
C_0178	Homocarnosine	ND	ND	ND	ND	ND	ND	ND	ND	ND	[illegible]
C_0148	Homocitrulline	6.96E-05	ND	0.000145	4.1E-05	0.000113	ND	5.72E-05	7.27E-05	0.000133	[illegible]
C_0189	Homocystine	ND	ND	ND	ND	ND	ND	ND	5.55E-05	ND	[illegible]
C_0043	Homoserine	ND	ND	ND	ND	ND	ND	ND	ND	ND	[illegible]
A_0076	Homovanillic acid	0.000178	0.000225	0.000169	0.000194	0.000318	0.000234	0.000174	0.000218	0.00026	[illegible]
C_0117	Hordenine	ND	ND	ND	ND	ND	ND	ND	ND	ND	[illegible]
C_0064	Hydroxyproline	0.002833	0.004487	0.002097	0.002405	0.003237	0.00189	0.002884	0.00212	0.001647	[illegible]
C_0031	Hypotaurine	0.000303	0.000172	0.000289	ND	0.000527	0.000182	6.88E-05	0.000299	0.000362	[illegible]
C_0077	Hypoxanthine	0.001944	0.001041	0.001454	0.001264	0.000946	0.001647	0.000743	0.000896	0.000602	[illegible]
A_0094	Ibuprofen	ND	ND	ND	ND	0.000136	ND	ND	ND	ND	[illegible]
C_0068	Ile	0.022352	0.045112	0.033376	0.037326	0.071194	0.025601	0.032413	0.039458	0.049436	[illegible]
C_0052	Imidazole-4-acetic acid	ND	ND	ND	ND	2.06E-05	ND	2.69E-05	ND	ND	[illegible]
C_0105	Imidazolelactic acid	5.49E-05	8.46E-05	0.000103	4.22E-05	0.000118	8.45E-05	7.03E-05	4.97E-05	0.000101	[illegible]
A_0071	Indole-3-acetic acid	ND	0.000143	0.000162	0.000205	0.002361	0.000158	ND	9.31E-05	0.000101	[illegible]
C_0190	Inosine	0.001083	0.000294	0.000578	0.000372	0.000506	0.001405	0.000323	0.000477	0.00239	[illegible]
A_0025	Isethionic acid	0.000265	0.000254	0.000445	0.00044	0.000579	0.000295	0.000279	0.000319	0.000449	[illegible]
A_0004	Isobutyric acidButyric acid	0.000603	0.000723	0.000895	0.000784	0.00086	0.000669	0.0007	0.000633	ND	[illegible]
C_0173	Isobutyrylcarnitine	ND	0.00021	ND	0.000213	0.000241	ND	0.000193	0.000347	0.000439	[illegible]
A_0084	Isocitric acid	0.001855	ND	0.002616	0.00201	0.00227	ND	ND	0.00156	0.002622	[illegible]
C_0081	Isoniazid	0.001796	0.000434	0.000668	0.002645	0.00023	ND	ND	ND	ND	[illegible]
C_0049	Isonicotinic acid	0.00717	0.009519	0.013549	0.003436	0.006654	ND	0.000193	ND	ND	[illegible]
C_0009	Isopropanolamine	ND	6.7E-05	9.75E-05	7.07E-05	0.000109	ND	9.4E-05	9.62E-05	ND	[illegible]
A_0010	Isovaleric acidValeric acid	ND	0.000209	0.000202	0.000297	0.000234	ND	ND	ND	0.00031	[illegible]
C_0182	Isovalerylcarnitine	ND	9.16E-05	ND	0.000121	0.000162	0.000134	ND	ND	ND	[illegible]
C_0160	Kynurenine	0.000363	0.000605	0.000551	0.000344	0.000896	0.000456	0.000735	0.000378	0.000478	[illegible]
A_0006	Lactic acid	0.182537	0.139333	0.117651	0.143483	0.109552	0.192085	0.08651	0.10098	0.119039	[illegible]
A_0091	Lauric acid	0.000506	0.000379	0.000537	0.000517	0.00041	0.000374	0.000371	0.000413	0.000373	[illegible]
C_0208	Lauroylcarnitine	ND	ND	6.27E-05	ND	ND	ND	ND	ND	ND	[illegible]
C_0067	Leu	0.047962	0.081431	0.073362	0.086263	0.127587	0.056857	0.067005	0.077274	0.099885	[illegible]
C_0176	Lidocaine	ND	ND	ND	ND	ND	ND	ND	ND	ND	[illegible]
C_0093	Lys	0.036272	0.05333	0.037716	0.048977	0.092351	0.055243	0.034796	0.073855	0.052971	[illegible]
A_0038	Malic acid	0.000848	0.001147	0.001455	0.001067	0.00127	0.001372	0.001115	0.000883	0.000867	[illegible]
C_0134	Mannosamine	0.000667	0.000385	0.00013	0.000109	0.000353	ND	ND	6.74E-05	ND	[illegible]
C_0054	Melamine	ND	ND	ND	ND	ND	ND	ND	ND	ND	[illegible]
C_0102	Mesalamine	8.63E-05	ND	7.5E-05	ND	ND	ND	ND	8.4E-05	ND	[illegible]
C_0096	Met	0.003827	0.006023	0.004082	0.004365	0.007228	0.003266	0.002183	0.005638	0.00421	[illegible]
C_0060	Metformin	ND	ND	ND	ND	ND	ND	ND	ND	ND	[illegible]
C_0114	Methionine sulfoxide	0.000369	0.000673	0.000306	0.00077	0.000678	0.000557	0.000501	0.000476	0.000676	[illegible]
C_0005	Methylguanidine	ND	ND	ND	ND	ND	ND	ND	ND	ND	[illegible]
C_0121	Metronidazole	ND	ND	ND	ND	ND	ND	ND	ND	ND	[illegible]
C_0170	Minocycline_divalent	ND	ND	ND	ND	ND	ND	ND	ND	ND	[illegible]
C_0012	Morpholine	ND	ND	ND	ND	ND	ND	ND	ND	ND	[illegible]
A_0096	Mucic acid	0.000669	0.000325	0.001051	0.001115	0.000333	0.000442	0.000632	0.000416	0.000419	[illegible]
A_0100	Myristoleic acid	ND	ND	7.89E-05	ND	ND	ND	ND	ND	ND	[illegible]
C_0023	N,N-Dimethylglycine	0.001454	0.001583	0.001676	0.001252	0.001722	0.001409	0.001711	0.001523	0.002205	[illegible]
A_0033	N-Acetyl-β-alanine	8.84E-05	0.000121	0.000156	ND	0.000104	9.4E-05	ND	0.000224	9.93E-05	[illegible]
A_0032	N-Acetylalanine	0.000215	0.000219	0.000482	0.000257	0.000339	0.000226	0.000145	0.000234	0.000315	[illegible]
A_0070	N-Acetylaspartic acid	ND	ND	ND	ND	ND	ND	ND	ND	ND	[illegible]
C_0165	N-Acetylgalactosamine-1<br>N-Acetylmannosamine-1<br>N-Acetylglucosamine-1	0.000244	0.000183	0.000266	0.000131	0.000329	0.00015	0.000125	0.000139	0.000238	[illegible]
C_0166	N-Acetylgalactosamine-2<br>N-Acetylmannosamine-2<br>N-Acetylglucosamine-2	ND	ND	ND	ND	ND	ND	ND	ND	0.000385	[illegible]
C_0164	N-Acetylglucosylamine	ND	ND	ND	ND	ND	ND	ND	ND	ND	[illegible]
A_0021	N-Acetylglycine	0.00025	0.000172	0.000359	0.000366	0.00025	0.000177	0.000494	0.000238	0.000182	[illegible]
C_0153	N-Acetylhistidine	ND	ND	2.68E-05	ND	ND	ND	ND	ND	ND	[illegible]
C_0144	N-Acetyllysine	ND	ND	4.06E-05	ND	7.02E-05	ND	ND	ND	ND	[illegible]
C_0124	N-Acetylornithine	0.001235	0.001062	0.001724	0.001909	0.002021	0.001492	0.000717	0.000455	ND	[illegible]
C_0062	N-Acetylputrescine	0.000104	6.9E-05	0.000127	5.12E-05	9.05E-05	8.3E-05	6.13E-05	0.00012	8.07E-05	[illegible]
C_0094	N-Acetylserine	ND	ND	0.000271	ND	ND	ND	ND	ND	ND	[illegible]
C_0027	N-Ethylglycine	ND	ND	ND	ND	ND	ND	ND	ND	ND	[illegible]
C_0135	N-Methylnorsalsolinol	ND	ND	ND	ND	ND	ND	ND	ND	ND	[illegible]
C_0057	N-Methylproline	ND	ND	ND	ND	0.00045	0.00021	0.004631	ND	0.001744	[illegible]
C_0142	N1-Acetylspermidine	7.11E-05	5.31E-05	0.000134	8.65E-05	5.86E-05	6.4E-05	5.42E-05	3.05E-05	4.04E-05	[illegible]
C_0101	N1-Methyl-4-pyridone-5-carboxamide	ND	0.000137	ND	0.000336	0.000179	9.17E-05	0.000194	ND	0.000143	[illegible]
A_0110	N2-Phenylacetylglutamine	0.000417	0.000185	0.000684	0.000453	0.000746	0.000269	0.000335	0.000651	0.000209	[illegible]
C_0123	N5-Ethylglutamine	0.001628	ND	ND	0.000291	ND	ND	ND	0.000179	ND	[illegible]
C_0147	N6,N6,N6-Trimethyllysine	0.000237	0.000311	0.000324	0.000237	0.000985	0.000247	0.000326	0.000277	0.000325	[illegible]
C_0143	N6-Acetyllysine	0.000223	0.000396	0.000318	0.000147	0.00029	ND	0.000136	0.00021	0.000156	[illegible]
C_0108	N6-Methyllysine	0.000774	0.000955	0.000957	0.000786	0.001018	0.001009	0.003905	0.000633	0.001356	[illegible]
C_0141	N8-Acetylspermidine	2.39E-05	ND	3.99E-05	2.8E-05	3.51E-05	ND	2.11E-05	ND	ND	[illegible]
C_0048	Nicotinamide	9.82E-05	8.48E-05	8.96E-05	0.000128	ND	ND	6.33E-05	ND	ND	[illegible]
C_0112	Nicotine	ND	ND	ND	ND	ND	ND	ND	ND	ND	[illegible]
C_0145	Nω-Methylarginine	5.42E-05	3.75E-05	ND	4.75E-05	ND	ND	ND	ND	5.08E-05	[illegible]
C_0157	O-Acetylcarnitine	0.006166	0.005889	0.008978	0.00973	0.006146	0.006974	0.006951	0.003966	0.009864	[illegible]
C_0109	O-Acetylhomoserine2-Aminoadipic acid	0.000158	0.000267	0.000254	0.000222	0.000589	0.000169	0.00013	0.000149	0.000323	[illegible]
A_0056	o-Coumaric acidp-Coumaric acid	ND	ND	ND	ND	ND	ND	ND	ND	ND	[illegible]
A_0041	o-Hydroxybenzoic acid	0.000474	ND	ND	ND	ND	ND	ND	ND	ND	[illegible]
A_0088	o-Hydroxyhippuric acid	ND	ND	ND	ND	ND	ND	ND	ND	ND	[illegible]
A_0043	Octanoic acid	ND	0.00014	0.000192	ND	ND	ND	0.000168	8.87E-05	ND	[illegible]
C_0196	Octanoylcarnitine	9.5E-05	ND	0.000265	0.000225	0.000583	ND	8.14E-05	ND	0.000176	[illegible]
C_0183	Octopine	ND	ND	ND	ND	5.01E-05	ND	ND	ND	ND	[illegible]
C_0197	Ophthalmic acid	ND	ND	ND	ND	5.83E-05	ND	ND	ND	ND	[illegible]
C_0072	Ornithine	0.016275	0.022431	0.016401	0.024444	0.022582	0.016858	0.018636	0.023545	0.024523	[illegible]
A_0005	Oxamic acid	ND	ND	ND	ND	ND	ND	ND	ND	ND	[illegible]
C_0100	Oxypurinol	ND	ND	ND	ND	ND	ND	ND	ND	ND	[illegible]
A_0040	p-Hydroxybenzoic acid	ND	ND	ND	ND	ND	ND	ND	ND	ND	[illegible]
A_0050	p-Hydroxyphenylacetic acid-1<br>p-Anisic acid-1<br>o-Hydroxyphenylacetic acid-1<br>Mandelic acid-1	ND	ND	ND	ND	ND	0.000176	ND	ND	0.00016	[illegible]
A_0051	Phenoxyacetic acid<br>p-Hydroxyphenylacetic acid-2<br>p-Anisic acid-2<br>o-Hydroxyphenylacetic acid-2<br>Mandelic acid-2	ND	ND	0.000281	ND	0.000208	ND	ND	0.000214	ND	[illegible]
A_0075	p-Hydroxyphenylpyruvic acid	ND	ND	ND	ND	ND	ND	ND	ND	ND	[illegible]
A_0098	Pantothenic acid	ND	ND	ND	0.000124	ND	ND	ND	ND	ND	[illegible]
C_0136	Paraxanthine	0.001041	0.001465	0.000448	0.002585	0.003029	0.002318	0.001181	0.000528	0.000882	[illegible]
A_0053	Pelargonic acid	0.000247	0.000229	0.000239	0.000265	0.000298	0.000265	0.000322	0.000238	0.000204	[illegible]
A_0060	Perillic acid	ND	ND	0.000194	ND	ND	ND	ND	ND	ND	[illegible]
C_0116	Phe	0.036918	0.067692	0.052323	0.046898	0.074605	0.042108	0.038865	0.051796	0.082604	[illegible]
C_0140	Phosphorylcholine	ND	ND	ND	ND	ND	ND	ND	ND	ND	[illegible]
C_0050	Picolinic acid	ND	ND	ND	ND	3.51E-05	ND	ND	ND	ND	[illegible]
C_0056	Pipecolic acid	0.000959	0.001084	0.00046	0.000567	0.001871	0.000713	0.000503	0.001045	0.000692	[illegible]
C_0011	Piperidine	7.62E-05	ND	7.99E-05	6.84E-05	5.25E-05	5.07E-05	7.98E-05	ND	0.000176	[illegible]
C_0037	Pro	0.041412	0.079168	0.038997	0.064219	0.06397	0.048133	0.062136	0.045753	0.096886	[illegible]
C_0013	Putrescine	8.8E-05	ND	ND	9.24E-05	ND	ND	ND	ND	ND	[illegible]
C_0010	Pyridine	ND	ND	ND	ND	ND	ND	ND	ND	ND	[illegible]
C_0118	Pyridoxal	ND	2.02E-05	ND	ND	ND	ND	ND	ND	ND	[illegible]
A_0003	Pyruvic acid	0.003567	0.003451	0.002126	0.002895	0.002215	0.006856	0.003101	0.002684	0.003186	[illegible]
A_0085	Quinic acid	ND	0.000385	0.000109	0.000435	0.000295	0.000168	ND	ND	0.000528	[illegible]
C_0206	Ranitidine	ND	ND	ND	ND	ND	ND	ND	ND	ND	[illegible]
A_0102	Ribulose 5-phosphate	ND	ND	ND	ND	ND	ND	ND	ND	ND	[illegible]
C_0132	S-Carboxymethylcysteine	ND	ND	ND	ND	0.01595	ND	ND	ND	ND	[illegible]
C_0075	S-Methylcysteine	0.00076	0.000991	0.00139	0.000765	0.00088	0.000609	0.000571	0.000433	0.001171	[illegible]
C_0113	S-Methylmethionine	ND	ND	ND	ND	ND	ND	ND	3.74E-05	5.11E-05	[illegible]
A_0092	S-Sulfocysteine	ND	9.6E-05	ND	ND	ND	ND	ND	ND	ND	[illegible]
C_0016	Sarcosine	ND	0.001127	0.000759	0.000654	0.000991	0.000948	0.000646	0.000748	ND	[illegible]
C_0155	SDMA	0.000297	0.000193	0.000356	0.000221	0.000374	0.00024	0.000228	0.000189	0.000433	[illegible]
C_0029	Ser	0.022711	0.028085	0.026012	0.027232	0.025217	0.034941	0.022041	0.018505	0.017761	[illegible]
C_0175	Ser-Glu	6.15E-05	7.45E-05	0.000113	0.000205	0.000254	0.000101	ND	0.000119	3.77E-05	[illegible]
C_0129	Serotonin	8.69E-05	9.88E-05	7.67E-05	0.000103	5.74E-05	0.000281	3.52E-05	6.3E-05	3.07E-05	[illegible]
C_0088	Stachydrine	0.001258	0.000735	0.003332	0.000348	0.010417	0.001259	0.036066	0.000578	0.018828	[illegible]
A_0022	Succinic acid	0.000665	ND	0.000837	0.000692	0.00088	0.000777	ND	0.000587	0.000589	[illegible]
A_0109	Sulfotyrosine	7.74E-05	ND	ND	0.000164	0.000179	ND	ND	ND	0.00015	[illegible]
A_0048	Tartaric acid	ND	ND	ND	ND	0.002179	ND	ND	ND	ND	[illegible]
C_0051	Taurine	0.003653	0.003728	0.004802	0.005421	0.004149	0.003331	0.003858	0.002891	0.00374	[illegible]
A_0139	Taurocholic acid	ND	ND	ND	ND	0.000247	ND	ND	ND	ND	[illegible]
A_0058	Terephthalic acid	0.000574	0.000541	0.000502	0.000622	0.000609	0.000633	0.000579	0.000603	0.000549	[illegible]
C_0137	Theobromine	0.000482	0.000459	0.000523	0.000821	0.001252	0.000611	0.00116	ND	0.000319	[illegible]
C_0187	Thiamine	ND	ND	ND	ND	ND	ND	ND	1.83E-05	ND	[illegible]
C_0073	Thioproline	ND	3.04E-05	3.5E-05	ND	ND	ND	2.33E-05	ND	6.54E-05	[illegible]
C_0042	Thr	0.022085	0.04375	0.0252	0.026291	0.056878	0.032288	0.021776	0.023445	0.029995	[illegible]
A_0039	Threonic acid	0.001532	0.001689	0.001864	0.002929	0.001142	0.001373	0.002333	0.001209	0.002102	[illegible]
A_0008	Tiglic acid	ND	ND	ND	ND	ND	ND	ND	ND	ND	[illegible]

C_0061	trans-Glutaconic acid	ND	ND	ND	ND	ND	ND	ND	ND	ND	ND	ND	ND	ND	ND	[illegible]
C_0097	Triethanolamine	4.94E-05	1.45E-05	ND	4.75E-05	3.94E-05	4.25E-05	5.64E-05	4.79E-05	ND	ND	ND	ND	4.31E-05	ND	[illegible]
C_0080	Trigonelline	0.000119	0.000578	0.000525	0.000416	0.000629	0.000239	0.000201	0.000338	0.000636	0.001078	0.000402	0.000185	0.000519	0.003218	[illegible]
C_0199	Trimethoprim	0.008955	ND	0.003981	ND	0.006211	ND	ND	0.003164	0.003574	ND	ND	0.006169	ND	0.005331	[illegible]
C_0008	Trimethylamine N-oxide	0.002281	0.00119	0.002441	0.001577	0.010351	0.001027	0.003138	0.002319	0.004927	0.004853	0.001316	0.0023	0.002527	0.002616	[illegible]
A_0059	Tropic acid<br>3-Phenyllactic acid<br>3-(2-Hydroxyphenyl)propionic acid<br>m-Ethoxybenzoic acid<br>p-Methoxyphenylacetic acid	ND	ND	ND	ND	ND	ND	ND	ND	ND	ND	ND	ND	ND	0.000375	[illegible]
C_0158	Trp	0.009285	0.020463	0.012769	0.024384	0.024943	0.017001	0.013943	0.01302	0.014422	0.019455	0.0249	0.015393	0.018003	0.017524	[illegible]
C_0139	Tyr	0.013454	0.028208	0.01147	0.025595	0.025632	0.016804	0.01436	0.020154	0.017109	0.022501	0.033352	0.020783	0.025293	0.030892	[illegible]
C_0119	Tyr-Arg_divalent	ND	ND	ND	ND	ND	ND	ND	ND	ND	ND	ND	ND	ND	ND	[illegible]
C_0205	Tyr-Glu	ND	ND	ND	ND	ND	ND	ND	ND	7.33E-05	8.65E-05	ND	4.17E-05	3.98E-05	7.64E-05	[illegible]
C_0033	Uracil	8.2E-05	8.48E-05	9.05E-05	ND	ND	8.3E-05	4.76E-05	ND	ND	ND	5.84E-05	0.000148	ND	ND	[illegible]
C_0001	Urea	0.005543	0.006662	0.006912	0.006104	0.008051	0.007413	0.006543	0.007385	0.011542	0.006334	0.005669	0.005894	0.006702	0.005857	[illegible]
A_0062	Uric acid	0.017821	0.013852	0.026086	0.029095	0.04234	0.015045	0.029335	0.017452	0.03168	0.003323	0.034569	0.021706	0.044157	0.031728	[illegible]
C_0181	Uridine	0.001178	0.001006	0.001633	0.001467	0.001068	0.001302	0.000937	0.001028	0.001054	0.001088	0.001357	0.0017	0.001154	0.000906	[illegible]
C_0083	Urocanic acid	ND	ND	ND	4.14E-05	ND	ND	ND	ND	ND	ND	7.68E-05	ND	ND	ND	[illegible]
C_0039	Val	0.060048	0.105999	0.077675	0.10343	0.148386	0.073123	0.077498	0.096678	0.115693	0.088371	0.138973	0.080894	0.10197	0.112412	[illegible]
A_0063	Vanillic acid	ND	ND	ND	ND	ND	ND	ND	ND	ND	ND	ND	ND	ND	ND	[illegible]
C_0211	Verapamil	ND	ND	ND	ND	ND	ND	ND	ND	ND	ND	ND	ND	ND	ND	[illegible]
A_0015	XA0002	0.000676	ND	0.000643	0.001036	0.001853	ND	ND	0.001024	0.000742	0.000855	0.000914	ND	0.000971	0.001145	[illegible]
A_0026	XA0003	ND	ND	ND	ND	ND	ND	ND	ND	ND	ND	ND	ND	ND	ND	[illegible]
A_0044	XA0004	ND	ND	ND	ND	7.99E-05	ND	ND	ND	ND	ND	ND	ND	ND	ND	[illegible]
A_0068	XA0013	0.000265	0.000918	0.001392	0.001233	ND	0.001442	0.001702	ND	0.00311	0.000521	0.000827	0.001429	0.000258	0.001088	[illegible]
A_0080	XA0017	ND	ND	ND	ND	ND	ND	ND	ND	ND	ND	ND	ND	ND	ND	[illegible]
A_0082	XA0019	ND	ND	ND	ND	ND	ND	ND	ND	0.000389	0.001116	0.000262	0.000618	0.000472	0.000586	[illegible]
A_0101	XA0027	0.000152	ND	0.000252	0.000202	0.000163	9.59E-05	0.000155	ND	ND	0.000154	0.00018	0.000103	0.000154	0.000139	[illegible]
A_0104	XA0035	ND	ND	ND	0.000259	ND	ND	ND	ND	ND	ND	ND	ND	ND	ND	[illegible]
C_0099	Xanthine	0.000556	0.00062	0.000673	0.000704	0.000974	0.000696	ND	ND	ND	ND	0.000501	ND	ND	0.000372	[illegible]
C_0003	XC0001	5.76E-05	ND	ND	1.85E-05	ND	4.98E-05	6.27E-05	ND	9.65E-05	ND	6.42E-05	ND	2.65E-05	4.78E-05	[illegible]
C_0055	XC0016	0.001325	0.001638	0.007103	0.001919	0.003822	0.001973	0.003293	0.001039	0.004205	0.002385	0.006123	0.000864	0.007798	0.003274	[illegible]
C_0059	XC0017	3.55E-05	ND	ND	4.5E-05	ND	4.33E-05	3.92E-05	ND	ND	ND	ND	5.38E-05	ND	ND	[illegible]
C_0087	XC0029	9.9E-05	9.41E-05	0.000124	8.88E-05	0.000215	7.13E-05	0.000483	6.23E-05	0.000275	0.000227	0.000103	0.000179	0.000189	0.000282	[illegible]
C_0122	XC0040	ND	ND	ND	ND	ND	ND	ND	ND	3.41E-05	3.71E-05	ND	4.74E-05	2.92E-05	3.36E-05	[illegible]
C_0162	XC0061	0.000257	0.000584	0.000212	0.000343	0.000374	0.000346	0.00037	0.000328	0.000625	0.000496	0.000717	0.000355	0.000497	0.000798	[illegible]
C_0163	XC0065	7.69E-05	5.68E-05	7.49E-05	ND	0.000106	5.75E-05	7.36E-05	ND	6.61E-05	5.51E-05	3.97E-05	ND	6.71E-05	4.79E-05	[illegible]
C_0200	XC0120	0.000215	0.00016	0.000421	0.000159	0.001046	0.000476	0.000153	0.000419	0.001559	0.001719	0.002135	0.001629	0.002006	0.00176	[illegible]
C_0204	XC0126	4.7E-05	4.99E-05	ND	ND	ND	ND	ND	ND	ND	ND	ND	ND	ND	ND	[illegible]
C_0207	XC0132	ND	2.44E-05	3.87E-05	3.31E-05	5.98E-05	4.09E-05	4.1E-05	4.41E-05	4.15E-05	3.42E-05	ND	2.56E-05	4.89E-05	4.45E-05	[illegible]
C_0015	β-Ala	0.000893	0.000701	0.000647	0.000553	0.000584	0.000407	0.000433	0.000434	0.000489	0.000433	0.000838	0.000426	0.000856	0.000545	[illegible]
A_0023	β-Hydroxyisovaleric acid	0.000283	0.000459	0.000298	0.000301	0.000498	0.000209	0.000171	0.000294	0.000262	0.00021	0.000395	0.000253	0.000259	0.000342	[illegible]
C_0090	γ-Butyrobetaine	0.000681	0.000993	0.000932	0.000912	0.00087	0.001092	0.00078	0.000757	0.001103	0.000904	0.001854	0.00087	0.00151	0.001297	[illegible]
C_0174	γ-Glu-2-aminobutyric acid	ND	ND	ND	ND	5.16E-05	ND	ND	ND	ND	ND	ND	ND	ND	ND	[illegible]
C_0201	γ-Glu-Val-Gly	ND	ND	ND	ND	ND	ND	ND	ND	ND	ND	ND	ND	3.78E-05	ND	[illegible]

C: Cation mode, A: Anion mode, ND: Not Detected, NA: Not Available, SD: standard deviation, m/z mass-to-charge ratio.

control 13	control 13	control 13	control 13	control 13	control 13	control 13	control 13	control 13	control 14	control 14	control 14	control 14	control 14	control 14	control 14	control 20	control 20	control 20	control 20
ND	ND	ND	ND	ND	ND	ND	ND	ND	ND	ND	ND	ND	ND	ND	ND	ND	ND	ND	0.000239
ND	ND	ND	ND	ND	ND	ND	ND	ND	ND	ND	ND	ND	ND	ND	ND	ND	ND	ND	ND
0.000153	0.000124	0.000126	9.07E-05	0.000458	0.000385	9.95E-05	0.000121	0.00025	0.000168	0.000101	0.000108	8.26E-05	9.4E-05	8.59E-05	0.000119	0.000127	7.6E-05	0.000594	0.000148
5.22E-05	5.03E-05	5.98E-05	5.35E-05	5.84E-05	5.78E-05	4.74E-05	6.92E-05	6.44E-05	6.65E-05	5.86E-05	6.28E-05	4.74E-05	6.67E-05	6.34E-05	5.66E-05	6.93E-05	5.55E-05	5.68E-05	9.17E-05
0.002034	0.002048	0.001391	0.001699	0.01286	0.007259	0.001595	0.002111	0.009107	0.006507	0.002357	0.002468	0.002271	0.002056	0.001156	0.001526	0.001877	0.002582	0.017299	0.003476
6.63E-05	ND	ND	3.09E-05	8.96E-05	ND	3.07E-05	3.51E-05	4.64E-05	3.75E-05	7.36E-05	5.44E-05	0.000103	ND	3.16E-05	0.000134	ND	4.8E-05	8.34E-05	3.96E-05
ND	ND	ND	ND	ND	2.62E-05	ND	4.13E-05	ND	ND	ND	ND	3.61E-05	ND	5.66E-05	ND	ND	ND	ND	ND
ND	3.79E-05	8.12E-05	ND	ND	ND	ND	ND	ND	ND	5.29E-05	ND	ND	ND	ND	ND	4.99E-05	ND	ND	ND
ND	ND	ND	ND	ND	ND	ND	ND	ND	ND	ND	ND	ND	ND	ND	ND	ND	ND	ND	ND
0.000476	0.000339	0.000355	0.000299	0.000355	0.000343	0.000372	0.000335	0.000409	0.000375	0.000318	0.000341	0.00039	0.000365	0.000332	0.000485	0.000394	0.000283	0.00041	0.000453
ND	ND	ND	ND	ND	ND	ND	ND	ND	ND	ND	ND	ND	ND	ND	ND	ND	ND	ND	ND
ND	ND	ND	ND	ND	ND	ND	ND	ND	ND	ND	ND	ND	ND	ND	ND	ND	ND	ND	ND
0.008308	0.003312	0.004713	0.004212	0.00636	0.009027	0.004986	0.007607	0.006546	0.004001	0.005769	0.007594	0.009228	0.005368	0.00482	0.008646	0.003262	0.007329	0.007576	0.00529
ND	ND	ND	ND	0.000179	ND	ND	ND	ND	ND	ND	0.000162	ND	ND	0.0004	ND	ND	ND	ND	ND
ND	ND	ND	ND	ND	ND	0.000172	ND	ND	ND	ND	ND	ND	ND	ND	ND	ND	ND	ND	ND
0.005676	0.00288	0.002861	0.001892	0.003463	0.004396	0.003517	0.003706	0.003001	0.001865	0.002232	0.005864	0.004181	0.002861	0.002596	0.004622	0.002166	0.004581	0.004501	0.00323
0.000245	ND	ND	0.000358	ND	0.000464	ND	0.000434	ND	ND	ND	ND	0.000365	0.000276	ND	0.000397	0.0004	0.001369	0.00031	0.000348
0.000329	ND	0.000396	ND	ND	ND	ND	ND	ND	ND	ND	ND	ND	ND	ND	0.000213	ND	ND	0.000321	ND
ND	ND	ND	ND	ND	ND	ND	ND	ND	ND	ND	ND	ND	ND	ND	ND	ND	ND	ND	ND
0.000759	0.001195	0.000839	0.00059	0.000793	0.000947	0.001145	0.000743	0.001046	0.00069	0.000853	0.001125	0.000902	0.001145	0.0012	0.002446	0.000812	0.000822	0.000502	0.001087
ND	ND	ND	ND	ND	ND	ND	ND	ND	ND	ND	ND	ND	ND	ND	ND	ND	ND	ND	ND
ND	ND	ND	ND	ND	ND	ND	ND	ND	ND	ND	ND	4.86E-05	ND	ND	ND	ND	ND	ND	ND
0.001048	ND	ND	0.001305	ND	ND	0.000895	0.000891	0.001061	ND	0.001455	0.001046	ND	0.001096	ND	0.001059	0.001039	ND	0.001006	0.001102
0.001474	0.001461	0.001332	0.001308	0.001812	0.001919	0.002083	0.001788	0.00166	0.001629	0.001458	0.001863	0.002357	0.002097	0.001672	0.001984	0.001878	0.001186	0.001447	0.002259

[illegible]

9.51E-05	ND	ND	ND	ND	ND	ND	ND	ND	ND	ND	ND	ND	ND	ND	ND	ND	ND	ND	ND	ND	ND	ND	ND	ND	ND	ND	ND	ND	ND	ND	ND	ND	ND	ND	ND	ND	ND	ND	ND	ND	ND	ND	ND	0.000284	ND	ND	ND	ND	ND	ND	ND	ND	ND	ND	ND	ND	0.000142	ND	ND	ND	ND	ND	ND	ND	ND	ND	ND	0.000177	ND	NA	NA	0.000162	3.2E-05	0.000141	3.78E-05	0.000201	7.39E-05	0.000162	3.2E-05	0.000167	5.99E-05
3.8E-05	ND	ND	ND	2.59E-05	ND	2.19E-05	ND	3.53E-05	ND	ND	ND	ND	ND	ND	ND	ND	ND	7.49E-05	3.65E-05	4.75E-05	ND	3.99E-05	ND	ND	3.87E-05	ND	ND	ND	7.32E-05	ND	ND	ND	5.21E-05	4.75E-05	5.45E-05	6.27E-05	5.6E-05	3.29E-05	3.65E-05	4.37E-05	5.58E-05	ND	2.69E-05	ND	ND	6.02E-05	0.000104	ND	5.69E-05	ND	ND	ND	4.05E-05	ND	ND	ND	ND	ND	ND	ND	ND	ND	ND	ND	ND	ND	2.2E-05	ND	ND	4.15E-05	1.21E-05	5.09E-05	3.83E-05	3.7E-05	1.22E-05	5.06E-05	1.84E-05	4.48E-05	2.41E-05	4.51E-05	1.74E-05
0.000275	5.72E-05	0.000223	0.001477	0.000684	0.001011	0.000404	0.001266	0.000558	0.000165	0.000753	0.001196	0.000111	0.000282	0.001173	0.000329	0.000661	0.000102	ND	0.000393	4.33E-05	0.001255	0.001457	5.8E-05	0.000165	0.000168	7.95E-05	0.00389	0.000117	0.00053	0.001828	8.99E-05	0.000247	0.000333	0.000375	0.000114	0.000465	0.000154	0.000215	0.001209	0.000294	0.001901	0.000609	5.02E-05	0.001083	0.000333	0.000184	8.05E-05	0.000177	4.83E-05	0.000356	0.001648	0.000513	0.000377	0.000845	0.000648	0.000117	8.08E-05	0.000638	0.000152	0.000794	0.000412	0.000676	0.000246	0.000186	0.000442	0.000139	0.000333	0.000194	0.000137	0.00044	0.000512	0.00065	0.000713	0.000588	0.000597	0.000522	0.000662	0.000552	0.000632	0.000552	0.00063
ND	ND	ND	ND	ND	ND	ND	ND	ND	ND	ND	ND	0.002984	ND	ND	ND	ND	ND	ND	ND	ND	ND	ND	ND	ND	ND	ND	ND	ND	ND	0.00266	ND	ND	ND	ND	ND	ND	ND	ND	ND	ND	ND	0.002389	ND	ND	ND	ND	ND	ND	ND	ND	ND	ND	ND	ND	ND	ND	0.000554	ND	ND	ND	ND	ND	ND	ND	ND	ND	ND	ND	ND	0.005065	0.001982	0.003202	0.002176	0.002984	NA	0.001868	0.001146	0.003964	0.002254	0.002147	0.001089
0.001713	0.000908	0.001749	0.001596	0.006278	0.000563	0.003375	0.002802	0.001914	0.013455	0.003226	0.014971	0.00517	0.004667	0.000675	0.002108	0.001032	0.002022	0.001712	0.001718	0.00067	0.011054	0.001172	0.011343	0.004571	0.001291	0.001614	0.007018	0.002515	0.007917	0.000563	0.001071	0.000413	0.001814	0.000871	0.001023	0.00147	0.000633	0.00526	0.001766	0.003597	0.009788	0.002884	0.001377	0.0101	0.003059	0.003733	0.00264	0.004144	0.000731	0.001646	0.001738	0.001493	0.00481	0.002193	0.001905	0.010493	0.007978	0.005617	0.000639	0.004249	0.001316	0.004011	0.001533	0.006648	0.007857	0.003366	0.000807	0.002311	0.002391	0.00406	0.004723	0.003264	0.002467	0.003531	0.00338	0.003437	0.003043	0.00363	0.003682	0.00348	0.003187
0.000556	ND	ND	ND	ND	ND	ND	ND	0.000675	ND	ND	ND	ND	ND	ND	ND	ND	0.000536	ND	ND	ND	ND	0.000496	0.000954	ND	0.000609	ND	ND	ND	ND	ND	ND	ND	ND	ND	ND	0.000623	0.000375	ND	ND	0.000444	0.001004	0.000505	ND	0.000493	ND	ND	ND	ND	ND	ND	ND	ND	ND	ND	ND	ND	ND	0.000534	0.00043	ND	ND	ND	ND	0.000353	ND	ND	ND	ND	ND	0.000385	0.000169	0.000522	0.000111	0.001117	0.0016	0.000566	0.0002	0.000477	0.000144	0.000791	0.001037
0.017676	0.011611	0.013348	0.016898	0.019725	0.023826	0.022355	0.020795	0.023438	0.019316	0.015714	0.017781	0.025412	0.018081	0.020809	0.02544	0.017278	0.019127	0.021759	0.020817	0.012256	0.022192	0.018693	0.008482	0.027189	0.024412	0.014972	0.018391	0.020071	0.021981	0.016524	0.019324	0.017468	0.018318	0.01735	0.02465	0.013408	0.019569	0.023394	0.019263	0.016622	0.018657	0.020198	0.018762	0.016622	0.023514	0.021348	0.011546	0.017092	0.013922	0.016896	0.017802	0.020025	0.020701	0.013429	0.012773	0.021378	0.008422	0.015107	0.017671	0.013685	0.021025	0.013187	0.016438	0.009962	0.021787	0.02083	0.018392	0.017107	0.018284	0.017753	0.004486	0.016797	0.004077	0.019583	0.004616	0.017964	0.004	0.017237	0.004275	0.018709	0.004348
0.022225	0.016869	0.015954	0.021485	0.024381	0.028648	0.038343	0.024155	0.027524	0.024911	0.017524	0.026192	0.0278	0.020297	0.02441	0.036622	0.018553	0.022968	0.025465	0.025452	0.019799	0.024008	0.018816	0.017692	0.034064	0.0316	0.023197	0.021404	0.024266	0.030302	0.017967	0.022402	0.020504	0.022236	0.019484	0.033523	0.026051	0.021455	0.033305	0.024812	0.02074	0.017349	0.030614	0.031967	0.021976	0.032918	0.030708	0.018035	0.027937	0.016909	0.033906	0.025448	0.024025	0.02955	0.017554	0.013934	0.029161	0.019776	0.015097	0.017785	0.018874	0.028187	0.01996	0.024384	0.011345	0.020579	0.028893	0.015982	0.023602	0.022562	0.022333	0.00611	0.022241	0.0057	0.024052	0.006868	0.023502	0.005707	0.022283	0.005862	0.023755	0.006241
ND	ND	ND	ND	ND	ND	ND	ND	ND	ND	ND	ND	ND	ND	ND	ND	ND	ND	ND	ND	ND	ND	ND	ND	ND	ND	ND	ND	ND	ND	ND	ND	ND	ND	ND	ND	ND	ND	ND	ND	ND	ND	ND	ND	ND	ND	ND	ND	ND	ND	ND	ND	ND	ND	ND	ND	ND	ND	ND	ND	ND	ND	ND	ND	ND	5.98E-05	ND	ND	ND	ND	7.15E-05	8.33E-05	3.34E-05	NA	2.35E-05	2.78E-06	5.98E-05	NA	6.2E-05	7.07E-05	3.56E-05	2.11E-05
ND	ND	ND	ND	2.65E-05	ND	ND	ND	ND	ND	ND	ND	ND	ND	3.77E-05	ND	ND	ND	ND	ND	ND	ND	ND	ND	ND	ND	ND	4.99E-05	ND	ND	ND	3.44E-05	ND	ND	ND	ND	ND	ND	3.33E-05	2.98E-05	2.98E-05	ND	3.67E-05	5.24E-05	ND	ND	ND	ND	ND	ND	ND	ND	ND	ND	ND	ND	ND	ND	ND	ND	ND	8.43E-05	3.49E-05	ND	ND	ND	ND	ND	ND	ND	4.3E-05	1.97E-05	4.76E-05	1.54E-05	3.8E-05	7.97E-06	4.28E-05	1.75E-05	4.5E-05	1.77E-05	4.04E-05	1.35E-05
9.86E-05	6.89E-05	5.28E-05	4.85E-05	8.12E-05	ND	ND	ND	ND	4.69E-05	ND	8.83E-05	ND	ND	9.94E-05	5.13E-05	ND	ND	ND	ND	ND	ND	6.11E-05	ND	5.48E-05	6.15E-05	7.37E-05	ND	ND	ND	7.48E-05	ND	5.58E-05	0.000131	0.000104	7.79E-05	ND	ND	6.39E-05	ND	ND	6.67E-05	ND	4.81E-05	ND	7.73E-05	ND	ND	9.09E-05	9.8E-05	ND	8.93E-05	8.23E-05	ND	6.44E-05	ND	0.000112	8.64E-05	6.91E-05	9.94E-05	ND	ND	ND	ND	ND	9.87E-05	ND	ND	ND	ND	8.51E-05	2.16E-05	8.27E-05	2.94E-05	7.81E-05	1.87E-05	8.01E-05	2.06E-05	8.37E-05	2.63E-05	7.91E-05	1.94E-05
0.006208	0.004534	0.005691	0.008321	0.006781	0.007716	0.006895	0.006084	0.005681	0.0057	0.004444	0.006008	0.00533	0.006868	0.006252	0.00753	0.004686	0.006635	0.007563	0.005161	0.0033	0.00558	0.006725	0.009835	0.009658	0.0069	0.005345	0.007487	0.005779	0.004382	0.004206	0.007069	0.005762	0.006598	0.004303	0.00437	0.00715	0.005043	0.007143	0.006037	0.003413	0.006098	0.00738	0.006828	0.008404	0.004614	0.00602	0.008958	0.008234	0.003586	0.005958	0.006679	0.003798	0.010734	0.006749	0.005031	0.006704	0.005887	0.007243	0.0051	0.009853	0.004827	0.00536	0.006592	0.006848	0.004869	0.006713	0.006354	0.006432	0.007204	0.006718	0.001654	0.006773	0.002026	0.006553	0.001363	0.006281	0.001665	0.006748	0.001855	0.006406	0.001532
0.015176	0.014681	0.021744	0.021987	0.019412	0.031846	0.021389	0.025077	0.020218	0.019112	0.026628	0.034514	0.028108	0.025318	0.016728	0.029022	0.020681	0.017961	0.033297	0.036769	0.014572	0.02444	0.029132	0.034429	0.043678	0.02046	0.015584	0.026332	0.016309	0.026907	0.015905	0.018408	0.019101	0.016159	0.029021	0.016313	0.013856	0.0208	0.023448	0.016987	0.024162	0.027084	0.03788	0.02377	0.020853	0.019369	0.019495	0.017914	0.026035	0.011794	0.020719	0.024744	0.016844	0.026976	0.017794	0.025605	0.017935	0.033045	0.028228	0.026505	0.028848	0.021627	0.030147	0.018195	0.024769	0.030181	0.017998	0.029101	0.029029	0.026184	0.025569	0.008079	0.026461	0.006408	0.024223	0.00679	0.023581	0.006751	0.026051	0.007201	0.023876	0.006742
0.0013	0.001034	0.001128	0.000955	0.001175	0.000758	0.001041	0.000946	0.000841	0.000923	0.001253	0.001339	0.001193	0.001177	0.001051	0.00098	0.000904	0.000856	0.001094	0.001255	0.001609	0.000904	0.001035	0.002477	0.001336	0.000966	0.001237	0.001183	0.000915	0.000848	0.000962	0.001263	0.000931	0.00153	0.001522	0.001164	0.000892	0.001028	0.001197	0.001006	0.000931	0.001134	0.001043	0.000946	0.001061	0.001423	0.00103	0.001214	0.00133	0.000971	0.000976	0.001037	0.001371	0.001064	0.001068	0.000853	0.001328	0.001242	0.001169	0.001108	0.001818	0.001179	0.001059	0.001284	0.001649	0.001119	0.000928	0.001352	0.001003	0.000937	0.001239	0.000256	0.001352	0.000324	0.001157	0.000224	0.001162	0.000284	0.0013	0.000299	0.00116	0.000257
7.62E-05	ND	4.8E-05	ND	ND	ND	ND	ND	ND	4.9E-05	ND	ND	ND	4.91E-05	ND	ND	ND	ND	ND	ND	ND	ND	ND	0.000246	ND	ND	ND	ND	5.01E-05	ND	ND	ND	ND	ND	ND	ND	ND	ND	ND	ND	ND	ND	ND	5.11E-05	ND	ND	ND	ND	ND	ND	4.43E-05	ND	ND	ND	ND	ND	ND	ND	ND	ND	ND	ND	6.96E-05	ND	ND	ND	ND	ND	ND	ND	8.11E-05	3.35E-05	7.97E-05	4.23E-05	7.45E-05	2.92E-05	9.23E-05	8.66E-05	8.01E-05	3.91E-05	7.95E-05	4.94E-05
0.101871	0.073062	0.078718	0.103591	0.118048	0.151877	0.117115	0.105862	0.117803	0.094753	0.076559	0.117866	0.117044	0.12897	0.093272	0.138059	0.093496	0.08617	0.137322	0.11329	0.065302	0.103539	0.121568	0.07194	0.144034	0.122115	0.093612	0.129335	0.087369	0.122475	0.079255	0.117208	0.098764	0.114389	0.099207	0.147939	0.103207	0.108305	0.132206	0.134134	0.089581	0.095171	0.145284	0.129856	0.109498	0.121509	0.120027	0.084155	0.102347	0.096833	0.104776	0.111355	0.09469	0.13621	0.094933	0.088216	0.113075	0.085526	0.101911	0.090978	0.112015	0.092548	0.114081	0.117152	0.06763	0.123335	0.124113	0.106103	0.096751	0.113563	0.102918	0.021355	0.103685	0.021807	0.110216	0.022634	0.107581	0.019518	0.103332	0.021495	0.108793	0.020942
ND	ND	ND	ND	ND	ND	ND	ND	ND	ND	ND	ND	ND	ND	ND	ND	ND	ND	ND	ND	ND	ND	ND	ND	ND	ND	ND	ND	ND	ND	ND	ND	ND	ND	ND	ND	ND	ND	ND	ND	ND	ND	ND	ND	ND	ND	ND	ND	ND	ND	ND	ND	ND	ND	ND	ND	ND	ND	ND	ND	ND	ND	ND	ND	ND	ND	ND	ND	ND	ND	0.007192	0.002258	0.001091	0.000872	0.000469	0.000288	NA	NA	0.003531	0.003581	0.000469	0.000288
ND	ND	ND	ND	ND	ND	ND	ND	ND	ND	ND	ND	ND	ND	ND	ND	ND	ND	ND	ND	ND	ND	ND	ND	ND	ND	ND	ND	ND	ND	ND	ND	ND	ND	ND	ND	ND	ND	ND	ND	ND	ND	ND	ND	ND	ND	ND	ND	ND	ND	ND	ND	ND	ND	ND	ND	ND	ND	ND	ND	ND	ND	ND	ND	ND	ND	ND	ND	ND	ND	NA	NA	9.6E-05	NA	NA	NA	NA	NA	9.6E-05	NA	NA	NA
0.001316	0.001099	0.001925	0.001058	0.001057	0.001386	ND	0.001534	0.000922	0.000915	0.001498	0.00119	ND	0.000817	ND	0.001031	0.000934	0.001378	0.000965	0.000944	0.000744	0.001186	ND	0.001276	0.000748	0.001565	0.00114	0.000953	ND	0.001159	ND	ND	ND	0.0007	ND	ND	ND	ND	ND	0.000944	ND	ND	ND	0.001192	0.000727	ND	0.001034	0.001345	0.001078	0.001186	ND	0.000889	0.000936	ND	ND	0.001432	0.001339	ND	ND	0.001182	ND	ND	ND	0.001223	ND	0.001127	ND	0.001413	0.00098	0.001165	0.001105	0.000388	0.0011	0.000412	0.001168	0.000365	0.001096	0.000224	0.001103	0.000399	0.001132	0.000302
ND	ND	ND	ND	ND	ND	ND	ND	ND	ND	ND	ND	ND	ND	ND	ND	ND	ND	ND	ND	ND	ND	ND	ND	ND	ND	ND	ND	ND	ND	ND	ND	ND	ND	ND	ND	ND	ND	ND	ND	ND	ND	ND	ND	ND	ND	ND	ND	ND	ND	ND	ND	ND	ND	ND	ND	ND	ND	ND	ND	ND	ND	ND	ND	ND	ND	ND	ND	ND	ND	0.000947	NA	0.000198	NA	0.000138	NA	NA	NA	0.000572	0.00053	0.000138	NA
8.07E-05	ND	ND	ND	ND	ND	9.86E-05	ND	ND	ND	ND	ND	ND	ND	ND	0.000127	ND	9.38E-05	ND	ND	9.47E-05	ND	ND	0.000187	ND	0.000114	ND	0.000118	ND	ND	ND	ND	ND	ND	ND	ND	ND	ND	ND	ND	ND	8.06E-05	ND	ND	ND	ND	ND	ND	ND	ND	ND	ND	ND	0.000167	0.000142	ND	ND	ND	ND	ND	0.000142	8.77E-05	ND	7.53E-05	ND	ND	ND	0.000169	0.000156	ND	0.000111	2.42E-05	0.000126	4.76E-05	0.000104	3.14E-05	0.000125	3.76E-05	0.000121	4.11E-05	0.000116	3.57E-05
0.00214	0.002183	0.000186	0.002417	0.003047	0.000634	0.004218	0.00107	0.000954	0.00285	0.000911	0.002375	0.000705	0.004708	0.001346	0.002191	0.000291	0.002903	0.000892	0.000636	0.00164	0.000543	0.000642	0.00388	0.002439	0.001134	0.001896	0.002042	0.005093	0.001246	0.000682	0.000854	ND	0.001471	0.000746	0.001381	0.000539	0.000746	0.000557	0.00025	0.002391	0.00338	0.007029	0.004153	0.01643	0.000832	0.000709	ND	0.006301	0.003202	0.000566	0.002096	0.000303	0.001377	0.001327	0.002929	0.001079	0.001636	0.007426	0.00111	0.004066	0.001317	0.002941	0.000527	0.001057	0.006359	0.000185	0.000493	0.001568	0.002648	0.001623	0.001605	0.002125	0.002304	0.002095	0.002345	0.002268	0.002696	0.001899	0.002026	0.002188	0.002528
ND	ND	ND	ND	ND	ND	ND	ND	ND	ND	ND	ND	ND	ND	ND	ND	ND	ND	ND	ND	ND	ND	ND	ND	ND	ND	ND	ND	ND	ND	ND	ND	ND	ND	ND	ND	ND	ND	ND	ND	ND	ND	ND	ND	ND	ND	ND	ND	ND	ND	ND	ND	ND	ND	ND	ND	ND	ND	ND	ND	ND	ND	ND	ND	ND	ND	ND	ND	ND	ND	0.00011	1.68E-05	0.000138	3.44E-05	NA	NA	NA	NA	0.000124	2.89E-05	NA	NA
0.000475	ND	ND	ND	ND	0.000467	ND	ND	ND	0.00057	ND	ND	ND	ND	ND	ND	ND	ND	ND	ND	ND	ND	ND	ND	0.000327	0.000414	ND	ND	ND	0.000392	0.000572	ND	ND	ND	ND	ND	0.000552	ND	ND	ND	ND	ND	ND	0.000473	ND	0.00049	ND	ND	ND	ND	ND	0.000295	ND	0.00066	ND	ND	ND	ND	0.000432	ND	ND	0.000695	0.000472	ND	ND	ND	ND	ND	ND	ND	0.000574	0.000244	0.000572	0.000309	0.000537	0.000377	0.000481	0.000122	0.000573	0.000278	0.000504	0.000248
0.000258	0.000225	0.000241	ND	0.000159	0.000157	0.000167	0.000237	0.000225	0.000158	0.000161	0.000175	0.000247	0.000194	0.00013	0.000169	0.000194	0.000179	0.000169	0.000181	0.000189	0.000131	0.00018	0.000305	0.000198	ND	0.000174	0.000236	ND	0.000173	ND	ND	0.000137	0.000151	0.000148	ND	ND	ND	0.000174	0.000225	0.000126	0.000137	0.000156	ND	ND	ND	0.000158	ND	0.000157	ND	ND	ND	0.00012	ND	ND	0.000201	ND	ND	0.000551	0.000155	ND	ND	0.00016	0.000115	ND	ND	0.000145	0.000184	ND	0.000188	0.000182	5.95E-05	0.000187	4.63E-05	0.000189	4.23E-05	0.000184	7.78E-05	0.000185	5.25E-05	0.000187	6.07E-05
0.000126	ND	ND	ND	0.000153	ND	ND	ND	ND	ND	ND	ND	ND	ND	ND	ND	ND	ND	ND	ND	ND	ND	ND	0.00014	ND	ND	ND	ND	ND	ND	ND	ND	ND	ND	ND	ND	ND	ND	ND	ND	ND	ND	ND	ND	ND	ND	ND	ND	ND	ND	ND	ND	ND	ND	ND	ND	ND	ND	0.000229	ND	ND	ND	ND	ND	ND	ND	ND	ND	ND	ND	0.000304	0.00028	0.000196	4.06E-05	0.000164	4.49E-05	0.000184	6.26E-05	0.000256	0.000208	0.000172	4.6E-05
ND	ND	ND	0.000501	ND	0.00068	ND	0.000691	0.000598	ND	ND	0.000634	ND	ND	ND	0.000834	ND	ND	ND	0.000867	ND	ND	0.000722	0.00059	ND	ND	0.0008	0.000966	0.000528	ND	0.000654	0.001346	0.000569	ND	0.000592	ND	ND	0.000654	0.000592	ND	ND	ND	ND	0.00043	0.000634	ND	ND	0.00047	0.000539	0.000487	ND	ND	ND	ND	0.000547	0.000643	ND	0.000634	0.000642	ND	0.004713	ND	ND	0.000705	0.001364	ND	ND	0.000629	ND	0.000483	0.000737	0.000305	0.000864	0.00081	0.000691	0.000153	0.000838	0.000823	0.000806	0.000628	0.000769	0.000608
5.55E-05	6.57E-05	ND	0.00015	8.15E-05	ND	0.000104	5.67E-05	6.16E-05	ND	ND	0.000119	5.57E-05	6.47E-05	7.06E-05	ND	4.04E-05	ND	ND	3.6E-05	4.14E-05	ND	ND	0.000267	4.79E-05	9.5E-05	ND	0.000126	0.000144	0.000152	ND	7.5E-05	ND	ND	4.06E-05	0.000444	ND	ND	ND	4.02E-05	0.000111	8.78E-05	7.15E-05	0.000133	4.23E-05	5.11E-05	ND	8.63E-05	ND	2.79E-05	ND	ND	ND	ND	ND	8.33E-05	ND	5.09E-05	5.83E-05	4.39E-05	0.000181	ND	ND	ND	ND	ND	5.91E-05	ND	9.96E-05	ND	6.62E-05	4.08E-05	7.26E-05	3.87E-05	8.54E-05	6.99E-05	9.77E-05	8.65E-05	6.99E-05	3.94E-05	9.17E-05	7.83E-05
0.001394	0.001989	0.002331	0.002394	0.001823	0.007224	0.002062	0.001571	0.004245	0.001271	0.001833	0.006535	0.004468	0.003816	0.003562	0.003767	0.004312	0.001857	0.009915	0.008237	0.001902	0.002156	0.005391	0.002883	0.004594	0.004648	0.001263	0.006067	0.002876	0.002417	0.001186	0.001134	0.000992	0.003402	0.002352	0.002742	0.001965	0.001375	0.002188	0.001796	0.005539	0.005141	0.009053	0.001189	0.002261	0.002086	0.003975	0.002334	0.004634	0.000796	0.006704	0.003592	0.001674	0.002365	0.001453	0.002431	0.00178	0.001117	0.001264	0.001692	0.019136	0.002118	0.002308	0.002165	0.005944	0.001165	0.003278	0.001932	0.011419	0.001867	0.003516	0.002725	0.003625	0.002707	0.003854	0.002599	0.00352	0.003205	0.003575	0.002702	0.003673	0.002932
4.84E-05	ND	6.74E-05	ND	ND	ND	ND	ND	ND	ND	ND	ND	ND	ND	ND	ND	ND	ND	ND	ND	ND	ND	ND	5.17E-05	ND	4.68E-05	ND	ND	4.11E-05	ND	ND	ND	4.71E-05	6.43E-05	ND	ND	ND	ND	5.04E-05	ND	1.88E-05	3.91E-05	ND	5.19E-05	ND	ND	4.94E-05	ND	ND	ND	5.81E-05	ND	ND	ND	ND	ND	ND	2.48E-05	ND	4.09E-05	ND	ND	ND	ND	ND	ND	6.08E-05	ND	4.88E-05	ND	4.76E-05	7.83E-06	4.87E-05	1.47E-05	4.51E-05	1.12E-05	4.63E-05	1.22E-05	4.81E-05	1.1E-05	4.59E-05	1.17E-05
0.000402	8.14E-05	9.31E-05	0.000154	0.000334	0.000251	0.000182	ND	0.000203	8.44E-05	0.000104	0.000157	0.00022	ND	0.000181	0.000148	0.000111	0.000106	0.000351	0.00026	0.000262	5.84E-05	0.000311	0.000438	0.000178	0.000189	0.00025	0.000233	8.86E-05	0.000162	0.000347	7.46E-05	0.0002	0.000205	5.45E-05	5.72E-05	0.000153	0.000117	9.78E-05	0.000143	0.000279	0.000197	0.000362	7.62E-05	0.000152	8.44E-05	0.00011	3.71E-05	0.00022	5.84E-05	5.61E-05	0.000159	0.000127	0.000195	0.000156	0.000204	0.000182	0.000128	0.000236	ND	0.001201	0.000202	0.00033	7.52E-05	0.000121	ND	0.000304	0.00025	0.000155	0.000106	0.000171	0.000113	0.000219	0.000161	0.000184	9.95E-05	0.000196	0.00017	0.000196	0.000142	0.000191	0.000143
ND	ND	ND	ND	5.11E-05	ND	ND	ND	ND	3.25E-05	3.19E-05	6.47E-05	ND	5.5E-05	5.92E-05	ND	ND	5.03E-05	ND	ND	ND	ND	ND	ND	ND	6.48E-05	ND	0.000115	ND	ND	ND	ND	ND	ND	ND	ND	ND	ND	ND	ND	ND	ND	ND	ND	7.21E-05	ND	ND	ND	ND	ND	ND	ND	ND	3.15E-05	4.48E-05	ND	ND	ND	ND	ND	ND	ND	ND	3.81E-05	ND	7.09E-05	4.44E-05	8.27E-05	ND	ND	4.57E-05	2.08E-05	4.84E-05	1.75E-05	4.53E-05	1.43E-05	6.14E-05	2.5E-05	4.71E-05	1.88E-05	5.12E-05	2.01E-05
0.000391	0.00028	0.000294	0.000559	0.000599	0.000493	0.000873	0.000539	0.000778	0.000446	0.00052	0.000922	0.000472	0.0008	0.000379	0.000696	0.000396	0.000576	0.000996	ND	0.000429	0.000614	0.000618	0.000621	0.00072	0.000521	0.000427	0.001144	0.000475	0.000644	0.000322	0.000434	0.000299	0.000533	0.000448	0.000449	0.000579	0.000396	0.000679	0.000515	0.000388	0.000518	0.000821	0.00057	0.001585	0.000867	0.000682	0.000742	0.000385	0.000426	0.000442	0.000493	ND	0.000628	0.000462	0.000688	0.000531	0.000249	0.000541	0.000434	0.000907	0.000428	0.000497	0.000865	0.000503	0.000568	0.000523	0.000448	0.000687	0.00068	0.000534	0.000175	0.00057	0.000221	0.000532	0.000175	0.000584	0.000226	0.000554	0.000201	0.00056	0.000205
7.03E-05	5.39E-05	7.08E-05	5.39E-05	5.06E-05	4.02E-05	6.9E-05	ND	5.46E-05	4.69E-05	6.26E-05	4.53E-05	4.69E-05	4.82E-05	4.7E-05	4.66E-05	ND	7.98E-05	6.24E-05	0.000128	7.78E-05	5.64E-05	ND	0.000185	0.000103	7.93E-05	6.96E-05	6.69E-05	5.54E-05	4.97E-05	ND	7.03E-05	ND	ND	ND	5.69E-05	6.36E-05	ND	5.82E-05	ND	6.22E-05	6.22E-05	ND	5.76E-05	ND	5.59E-05	5.21E-05	5.38E-05	ND	4.22E-05	8.59E-05	9.73E-05	3.43E-05	7.87E-05	3.83E-05	7.21E-05	4.47E-05	8.85E-05	6.95E-05	5.04E-05	0.00011	5.29E-05	ND	0.000111	6.67E-05	ND	ND	ND	8.31E-05	8.66E-05	7.14E-05	2.52E-05	7.39E-05	2.85E-05	6.53E-05	1.88E-05	7.23E-05	2.79E-05	7.28E-05	2.7E-05	6.87E-05	2.39E-05
0.000662	0.000357	0.001087	0.00026	0.000914	0.000624	0.000931	0.000823	0.000624	0.00094	0.000749	0.001452	0.000515	0.000411	0.000671	0.000679	0.00029	0.000527	0.000597	0.00058	0.000535	0.000579	0.00051	0.000702	0.000924	0.000955	0.000863	0.000792	0.000693	0.000602	0.000769	0.000655	0.000409	0.00022	0.001039	0.000464	0.000666	0.000422	0.000469	0.000514	0.000951	0.000792	0.000665	0.000561	0.000319	0.001079	0.00032	0.001112	0.00053	0.000461	0.000373	0.000989	0.000656	0.000745	0.000296	0.000645	0.00035	0.000778	0.000579	0.000173	0.00083	0.000567	0.001149	0.000572	0.0008	0.000545	0.000312	0.000419	0.000274	0.000669	0.000726	0.000549	0.000805	0.000543	0.000703	0.000305	0.000616	0.000238	0.000769	0.000544	0.000657	0.000273
ND	ND	ND	3.15E-05	ND	ND	ND	ND	ND	ND	3.09E-05	ND	ND	ND	ND	3.63E-05	ND	ND	ND	ND	ND	ND	ND	ND	ND	ND	ND	ND	ND	ND	ND	ND	ND	ND	ND	ND	ND	ND	ND	ND	ND	ND	ND	ND	3.79E-05	ND	ND	ND	4.56E-05	ND	ND	ND	ND	ND	ND	ND	ND	ND	ND	ND	7.88E-05	ND	ND	ND	ND	ND	ND	5.49E-05	3.18E-05	ND	5.69E-05	3E-05	4.92E-05	1.35E-05	4.19E-05	1.12E-05	4.98E-05	1.84E-05	5.04E-05	1.64E-05	4.55E-05	1.47E-05
4.77E-05	4.99E-05	5.48E-05	7.5E-05	3.76E-05	4.13E-05	5.02E-05	ND	3.51E-05	ND	2.84E-05	2.95E-05	ND	ND	ND	ND	ND	ND	3.74E-05	ND	5.7E-05	3.17E-05	4.28E-05	0.000163	5.65E-05	4.78E-05	ND	ND	4E-05	ND	ND	ND	ND	ND	ND	ND	ND	ND	3.81E-05	2.56E-05	ND	ND	ND	3.07E-05	ND	ND	3.92E-05	3.03E-05	ND	3.97E-05	3.61E-05	ND	ND	5.23E-05	ND	4.01E-05	ND	5.1E-05	ND	ND	ND	ND	3E-05	5.73E-05	8.06E-05	ND	ND	ND	3.88E-05	ND	4.31E-05	1.75E-05	5.26E-05	2.12E-05	4.57E-05	1.34E-05	4.84E-05	2.84E-05	4.81E-05	2E-05	4.69E-05	2.11E-05
0.000555	0.000936	0.000352	0.000537	0.000828	0.001095	0.000419	0.00105	0.000648	0.00152	0.00073	0.000663	0.000533	0.000548	0.000547	0.000782	0.000658	0.000697	0.000886	0.000671	0.000537	0.007918	0.000656	0.000629	0.000974	0.001468	0.000341	0.000953	0.000504	0.000497	0.000336	0.000594	0.000943	0.001426	0.000442	0.000511	0.000726	0.000481	0.000839	0.000538	0.000616	0.000451	0.001528	0.000711	0.000466	0.000556	0.002601	0.000466	0.00059	0.000306	0.00036	0.000342	0.000361	0.012448	0.000699	0.000328	0.000483	0.000439	0.000654	0.000515	0.00101	0.000759	0.000528	0.000697	0.000561	0.006002	0.000409	0.000429	0.00093	0.000781	0.005436	0.032332	0.000624	0.000245	0.000958	0.001156	0.001134	0.002017	0.002838	0.021932	0.001053	0.001671
0.000329	0.000171	0.000283	0.000166	0.000296	0.000464	0.000382	0.000343	0.000202	0.000338	0.00025	0.000285	0.000221	0.000354	0.000234	0.000468	0.000472	0.00029	0.000393	0.000346	0.000265	0.000242	0.000311	0.000349	0.000403	0.00015	0.000235	0.000439	0.000286	0.000325	0.000237	0.000232	0.000355	0.000576	0.000278	0.000314	0.00031	0.000264	0.000224	0.000464	0.000192	0.000306	0.000444	0.00027	0.000246	0.000181	0.000348	0.00024	0.000407	0.000251	0.000254	0.000309	0.000242	0.00032	0.000387	0.000272	0.000228	0.000117	0.000396	0.000366	0.000433	0.000296	0.000262	0.000319	0.00044	0.000247	0.000308	0.000317	0.000367	0.000345	0.00032	0.000141	0.000321	0.000115	0.000333	0.000125	0.000312	8.74E-05	0.000321	0.000127	0.000322	0.000106
0.002244	0.001111	0.000998	0.00095	0.001131	0.000845	0.00124	0.00132	0.000875	0.00099	0.001596	0.000909	0.000939	0.001216	0.001102	0.001045	0.000977	0.001051	0.001706	ND	0.001755	0.001079	0.000996	0.002938	0.001255	0.00076	0.000968	0.001581	0.000821	0.000706	0.00068	0.000841	0.001383	0.001056	0.000943	0.000975	0.000893	0.000734	0.001094	0.000863	0.000899	0.00109	0.001561	0.000864	0.001192	0.000838	0.001093	0.001277	0.001084	0.00064	0.000874	0.000951	0.000342	0.002133	0.001324	0.000735	0.001192	0.000748	0.000802	0.00078	0.002063	0.000832	0.000886	0.001023	0.000668	0.001191	0.000706	0.00088	0.001458	0.001105	0.001118	0.000328	0.00109	0.000355	0.001131	0.000283	0.001081	0.000435	0.001103	0.000341	0.001104	0.000371
ND	ND	ND	ND	ND	2.88E-05	ND	ND	ND	ND	ND	ND	ND	ND	ND	ND	ND	ND	3.49E-05	ND	ND	ND	ND	ND	ND	ND	ND	ND	ND	ND	ND	ND	ND	ND	ND	ND	ND	ND	ND	ND	ND	ND	ND	ND	5.19E-05	ND	ND	ND	ND	ND	ND	ND	ND	ND	ND	ND	ND	ND	ND	ND	ND	ND	ND	ND	ND	ND	ND	ND	ND	ND	5.73E-05	5.01E-06	4.48E-05	1.04E-05	3.36E-05	6.92E-06	4.34E-05	1.2E-05	5.02E-05	1.03E-05	3.85E-05	9.79E-06
ND	ND	ND	ND	ND	4.95E-05	ND	ND	ND	ND	ND	ND	ND	ND	ND	ND	ND	ND	ND	ND	ND	ND	ND	ND	ND	7.06E-05	ND	ND	ND	ND	ND	ND	ND	ND	ND	ND	ND	ND	6.23E-05	ND	ND	1.89E-05	ND	ND	ND	ND	ND	ND	ND	ND	ND	ND	ND	ND	ND	ND	ND	ND	ND	ND	ND	ND	ND	ND	ND	ND	ND	ND	ND	ND	4.01E-05	9.23E-06	3.9E-05	9.87E-06	5.7E-05	1.74E-05	5.06E-05	2.77E-05	3.96E-05	8.57E-06	5.38E-05	2.1E-05
