# Supplementary material for: Serum Metabolomic Profiling in Rheumatoid Arthritis Patients With Interstitial Lung Disease: A Case–Control Study
Source: Front Med (Lausanne). 2020 Dec 17;7:599794. doi: 10.3389/fmed.2020.599794 (PMC7773768; doi:10.3389/fmed.2020.599794)
Supplement: Supplementary file 2 [file Table_2.pdf]

Supplemental Table S2. Normalized metabolomic profile of detected metabolites

Compound name	case 101	case 102	case 103	case 104	case 105	case 106	case 107	case 108	case 109	case 110	case 111	case 112	case 113	case 114	case 115	case 116	case 117	case 118	case 119	case 120	case 121	case 122	case 123	case 124	case 125	case 126	case 127	case 128	case 129	case 130	case 131	case 132	case 133	case 134	case 135	case 136	case 137	case 138	case 139	case 140	case 141	case 142	case 143	case 144	case 145	case 146	case 201	case 202	case 203	case 204	case 205	case 206	case 207	case 208	case 209	case 210	case 211	case 212	case 213	case 214	case 215	case 216	case 217	case 218	case 219	case 220	case 221	case 222	case 223	case 224	case 225	case 226	case 227	case 228	case 229	case 230	case 231	case 232	case 233	case 234	case 235	case 236
Decanoic acid	-0.81413	-0.81413	-0.81413	-0.81413	-0.81413	-0.81413	-0.81413	-0.81413	-0.81413	-0.81413	-0.81413	-0.81413	-0.81413	-0.81413	-0.81413	-0.81413	-0.81413	-0.81413	-0.81413	-0.81413	-0.81413	-0.81413	0.123839	-0.81413	-0.81413	0.40022	-0.81413	-0.81413	-0.81413	-0.81413	-0.81413	1.666968	-0.81413	-0.81413	-0.81413	1.091173	0.40022	0.976015	-0.81413	-0.81413	-0.81413	-0.81413	1.091173	-0.81413	-0.81413	-0.81413	-0.81413	0.745697	-0.81413	-0.81413	1.321491	-0.81413	-0.81413	-0.81413	-0.81413	0.40022	-0.81413	-0.81413	-0.81413	0.630538	-0.81413	-0.81413	-0.81413	0.745697	-0.81413	1.551809	-0.81413	-0.81413	-0.81413	-0.81413	1.321491	-0.81413	-0.81413	-0.81413	-0.81413	-0.81413	-0.81413	-0.81413	-0.81413	-0.81413	-0.81413	-0.81413
Glycerol	1.160482	0.158833	0.921994	1.160482	0.158833	1.637458	1.160482	0.731204	-0.89051	-1.129	0.349623	1.637458	0.349623	1.01739	-1.51058	0.063438	0.731204	1.065087	1.065087	-0.12735	0.826599	1.01739	0.731204	1.637458	0.206531	-0.74742	0.111135	-0.41354	0.445019	0.349623	-0.31814	1.637458	-0.65203	-0.65203	1.637458	2.59141	0.01574	-0.03196	1.160482	0.969692	1.637458	-0.03196	0.349623	-0.22275	2.114434	-0.07965	1.160482	-1.51058	-0.69972	0.01574	0.301926	-0.17505	-1.27209	-0.50893	-0.22275	-0.03196	-0.07965	0.635809	0.588111	0.349623	0.349623	-0.79512	0.206531	-1.89216	-0.12735	0.158833	0.445019	0.492716	1.160482	0.158833	-0.69972	0.301926	2.59141	0.540414	-0.07965	-0.79512	0.158833	0.254228	0.969692	2.59141	1.112785	2.59141
Morpholine	-0.4295	-0.4295	-0.4295	-0.4295	-0.4295	-0.4295	-0.4295	-0.4295	-0.4295	-0.4295	-0.4295	-0.4295	-0.4295	-0.4295	-0.4295	-0.4295	-0.4295	-0.4295	-0.4295	-0.4295	-0.4295	-0.4295	-0.4295	-0.4295	-0.4295	-0.4295	-0.4295	-0.4295	-0.4295	-0.4295	-0.4295	-0.4295	-0.4295	-0.4295	-0.4295	-0.4295	-0.4295	-0.4295	-0.4295	-0.4295	-0.4295	-0.4295	-0.4295	-0.4295	-0.4295	-0.4295	-0.4295	-0.4295	-0.4295	-0.4295	-0.4295	-0.4295	-0.4295	-0.4295	-0.4295	-0.4295	-0.4295	-0.4295	-0.4295	-0.4295	-0.4295	-0.4295	-0.4295	-0.4295	-0.4295	-0.4295	-0.4295	-0.4295	-0.4295	-0.4295	-0.4295	-0.4295	-0.4295	-0.4295	-0.4295	-0.4295	-0.4295	-0.4295	-0.4295	-0.4295	-0.4295	-0.4295
Dyphylline	2.014157	0.172221	0.172221	0.7862	-0.44176	0.7862	0.172221	0.7862	-0.68735	-0.68735	0.7862	2.014157	-1.11713	0.172221	-1.05574	0.172221	0.172221	1.400178	1.400178	0.172221	0.7862	0.7862	1.400178	1.400178	0.172221	-0.74875	1.400178	-0.44176	-0.44176	0.7862	-0.44176	0.7862	-0.44176	-1.11713	2.014157	2.014157	0.172221	-0.44176	2.014157	1.400178	0.7862	-0.44176	0.172221	-0.44176	1.400178	-0.44176	1.400178	-1.30133	-0.44176	-0.81014	0.7862	-0.44176	-1.30133	-1.17853	0.172221	-0.44176	-0.44176	0.172221	0.7862	0.7862	0.7862	-0.62595	-0.81014	-2.1609	-0.44176	1.400178	1.400178	-0.44176	1.400178	0.7862	-0.68735	-0.44176	2.628135	0.7862	0.172221	0.7862	0.172221	0.172221	0.7862	1.400178	0.7862	1.400178
Octanoic acid	-1.21706	-0.17068	0.215576	-1.21706	-1.21706	-1.21706	0.061072	-0.56467	-1.21706	-1.21706	-0.09343	-1.21706	-1.21706	-1.21706	-1.21706	-1.21706	0.756341	-1.21706	-0.01618	-1.21706	0.988097	-0.51059	-1.21706	-1.21706	-1.21706	0.524584	-1.21706	0.215576	-1.21706	1.992375	0.524584	1.683366	1.528862	0.447332	0.601836	1.297105	0.138324	0.215576	-0.09343	-0.24794	0.292828	0.756341	-0.09343	-1.21706	-0.01618	0.215576	-0.09343	0.215576	0.37008	-1.21706	0.756341	-1.21706	-1.21706	-1.21706	-1.21706	0.292828	0.215576	-0.24794	1.528862	0.601836	-1.21706	-1.21706	0.061072	0.601836	0.138324	1.528862	-0.01618	-0.09343	1.142601	0.833593	2.069627	-1.21706	0.447332	-0.17068	-0.01618	-0.24794	-1.21706	-1.21706	0.292828	-1.21706	-1.21706	-1.21706
Fumaric acid monomethyl ester	-1.19193	-1.19193	-1.19193	-1.19193	-1.19193	-1.19193	-1.19193	-1.19193	-1.19193	-0.80028	-0.8384	-1.19193	-1.19193	-0.64777	-1.19193	-1.19193	-1.19193	-1.19193	-0.20932	-0.66684	-0.11401	-0.45714	0.896335	0.30538	0.248191	-0.30464	0.133813	0.896335	0.591326	-0.26651	-0.60965	0.076624	-0.22839	-0.38089	-0.1712	1.849489	-0.34276	-0.13307	0.229128	1.277597	-0.11401	-0.72403	1.468227	0.210065	0.286317	0.229128	-1.19193	-1.19193	-1.19193	-1.19193	-1.19193	-1.19193	-1.19193	-1.19193	-0.51433	-0.70496	-0.74309	-0.57152	-1.19193	-1.19193	-1.19193	-1.19193	-1.19193	0.267254	1.277597	0.038497	-0.20932	0.133813	-0.01869	0.038497	-0.15213	-0.30464	0.000371	-0.26651	-0.39995	-0.28557	-0.51433	-0.62871	0.591326	0.152876	-1.19193	-0.45714
N-Acetylgalactosamine-1																																																																																		
N-Acetylmannosamine-1	1.01114	-0.11131	1.572365	-1.04668	2.694815	-0.67253	-1.04668	-0.85961	1.01114	-0.11131	-0.11131	-0.29838	0.26284	0.075765	0.63699	-1.23376	1.01114	-1.23376	0.824065	1.572365	0.63699	1.01114	0.26284	-0.29838	-1.04668	0.26284	-0.11131	-0.11131	1.198215	0.075765	-0.48546	0.26284	0.824065	-0.48546	0.075765	2.50774	-0.85961	-0.11131	-0.67253	0.449915	0.075765	1.946515	0.26284	0.075765	-0.29838	-1.23376	-0.85961	-0.67253	-0.29838	-0.85961	0.449915	-1.04668	-1.04668	2.694815	-0.11131	-0.48546	-1.23376	0.449915	3.068965	-0.67253	-0.29838	-0.48546	-0.11131	-0.29838	-0.29838	1.75944	0.26284	0.449915	0.449915	1.572365	-0.48546	2.13359	1.38529	2.13359	1.198215	-0.11131	0.63699	3.25604	0.26284	0.26284	1.572365	0.63699
N-Acetylglucosamine-1																																																																																		
Trimethoprim	5.806202	-0.32643	2.397463	-0.32643	3.897308	-0.32643	-0.32643	1.852064	2.124764	-0.32643	-0.32643	3.897308	-0.32643	3.283735	-0.32643	-0.32643	-0.32643	-0.32643	-0.32643	-0.32643	-0.32643	-0.32643	-0.32643	-0.32643	-0.32643	-0.32643	-0.32643	-0.32643	-0.32643	-0.32643	-0.32643	-0.32643	-0.32643	-0.32643	-0.32643	-0.32643	-0.32643	-0.32643	-0.32643	-0.32643	-0.32643	1.374841	3.488259	-0.32643	-0.32643	-0.32643	2.465637	-0.32643	-0.32643	-0.24772	-0.32643	-0.32643	1.238491	3.897308	2.261113	-0.32643	0.556744	-0.32643	3.283735	-0.32643	1.51119	-0.32643	-0.11819	-0.32643	-0.32643	-0.32643	-0.32643	-0.32643	-0.32643	-0.32643	-0.32643	-0.32643	-0.32643	4.101832	-0.32643	0.147695	-0.32643	-0.32643	-0.32643	-0.32643	-0.32643	-0.32643
Melamine	-0.48062	-0.48062	-0.48062	-0.48062	-0.48062	-0.48062	-0.48062	-0.48062	-0.48062	-0.48062	-0.48062	-0.48062	-0.48062	-0.48062	1.848736	-0.48062	-0.48062	-0.48062	2.192679	1.848736	2.020708	-0.48062	-0.48062	-0.48062	-0.48062	1.504792	1.676764	1.504792	-0.48062	-0.48062	-0.48062	-0.48062	-0.48062	-0.48062	3.052539	2.364651	-0.48062	1.848736	-0.48062	2.364651	2.708595	-0.48062	2.192679	-0.48062	2.708595	1.676764	1.676764	-0.48062	-0.48062	1.676764	1.504792	-0.48062	-0.48062	2.192679	-0.48062	2.020708	-0.48062	-0.48062	-0.48062	-0.48062	-0.48062	-0.48062	-0.48062	2.192679	-0.48062	-0.48062	-0.48062	1.848736	-0.48062	-0.48062	-0.48062	2.536623	-0.48062	1.848736	-0.48062	-0.48062	-0.48062	2.192679	-0.48062	-0.48062	-0.48062	-0.48062
Uridine	-0.10164	-0.80022	1.295507	0.94622	-0.45093	0.247645	-1.00979	-0.80022	-0.45093	-0.45093	0.596932	1.644795	-0.10164	-1.11458	-0.10164	0.94622	0.247645	-0.10164	1.994083	0.596932	0.247645	3.041945	0.596932	0.596932	0.247645	-0.45093	0.596932	-0.45093	0.94622	-0.80022	-0.45093	-0.45093	0.247645	-1.70837	0.94622	-0.80022	-0.10164	-0.10164	-0.80022	-0.80022	-0.10164	-1.21936	1.295507	0.247645	-0.45093	-0.80022	-0.87008	-0.10164	1.994083	-0.10164	0.247645	-0.10164	-0.80022	-0.45093	0.94622	0.94622	0.247645	-0.10164	0.596932	-0.45093	-0.97486	-1.00979	-0.45093	1.295507	1.644795	0.94622	0.247645	0.596932	-0.45093	0.94622	-1.28922	2.692658	0.247645	1.994083	-0.10164	-0.10164	1.295507	0.247645	-0.45093	0.94622	0.596932	0.247645
8-Hydroxy-2'-deoxyguanosine	-0.61345	0.378126	1.253047	1.544688	0.086486	0.086486	-0.26348	0.669767	0.669767	0.348962	0.669767	0.232306	0.232306	-0.29265	-0.70094	0.961407	0.028158	-0.38014	0.961407	0.378126	0.290634	1.836328	-0.20515	-0.46763	-2.40558	1.544688	-2.40558	-2.40558	-2.40558	0.961407	0.669767	1.253047	0.669767	-0.61345	-0.4093	0.232306	-0.0885	0.669767	0.961407	1.836328	0.348962	0.669767	0.378126	1.253047	1.253047	0.669767	0.086486	-0.00101	-0.11766	0.203142	-0.11766	0.669767	-0.00101	-0.20515	0.057322	0.290634	0.290634	0.144814	1.253047	-0.78844	-0.05933	-0.78844	0.203142	-0.96342	0.319798	0.203142	0.961407	-0.00101	0.669767	0.378126	-0.78844	0.348962	1.836328	0.669767	0.961407	-0.46763	0.144814	0.669767	0.378126	0.961407	1.253047	1.253047
Ser-Glu	0.369944	0.598132	1.282694	3.184256	3.944881	1.092538	-0.7225	1.47285	-0.08643	-0.7225	-0.7225	-0.7225	-0.7225	0.236835	-0.21954	0.465022	2.9941	0.446007	-0.0484	-0.06742	0.350929	0.769272	4.135037	0.769272	0.978444	0.712225	0.065694	2.423631	0.902382	0.5601	-0.7225	-0.7225	-0.7225	-0.7225	-0.7225	0.046679	-0.7225	0.046679	-0.7225	-0.7225	-0.02938	-0.27659	0.331913	-0.7225	0.217819	-0.02938	-0.27659	-0.7225	-0.7225	-0.7225	-0.7225	1.092538	-0.7225	-0.33363	-0.7225	-0.23856	-0.39068	0.179788	-0.7225	-0.7225	1.47285	5.46613	1.663006	0.69321	-0.01037	1.282694	1.663006	0.25585	-0.7225	0.807303	1.47285	0.769272	-0.7225	-0.06742	0.826319	-0.23856	0.38896	-0.7225	-0.7225	-0.7225	-0.02938	0.103725

case 237	case 238	case 239	case 240	case 241	case 242	case 243	case 244	case 245	case 246	case 247	case 248	case 249
-0.81413	-0.81413	-0.81413	-0.81413	-0.81413	-0.81413	-0.81413	-0.81413	-0.81413	-0.81413	-0.81413	-0.81413	-0.81413
0.921994	0.778902	0.731204	1.160482	-0.50893	0.921994	2.59141	1.01739	2.59141	-0.31814	3.068386	0.206531	0.254228
-0.4295	-0.4295	-0.4295	-0.4295	-0.4295	-0.4295	-0.4295	-0.4295	-0.4295	-0.4295	-0.4295	-0.4295	-0.4295
1.400178	0.7862	1.400178	0.7862	-0.81014	-0.44176	1.400178	0.172221	2.014157	-0.44176	2.014157	2.014157	0.7862
-1.21706	-1.21706	-0.6342	0.138324	-1.21706	-1.21706	-1.21706	-1.21706	0.37008	-0.24794	-1.21706	-1.21706	-1.21706
0.896335	-0.59058	-0.51433	-0.01869	0.191002	-0.55246	0.229128	2.612012	-0.15213	0.05756	1.658858	0.705705	-0.1712
0.449915	1.946515	-0.48546	-0.11131	-1.42083	-1.23376	-0.29838	0.075765	1.198215	0.075765	1.75944	0.26284	1.572365
2.192938	-0.32643	-0.32643	-0.32643	-0.32643	-0.32643	-0.32643	-0.32643	-0.32643	2.806511	-0.32643	-0.32643	-0.32643
-0.48062	1.160849	1.504792	-0.48062	-0.48062	-0.48062	-0.48062	0.937285	-0.48062	-0.48062	2.536623	-0.48062	-0.48062
0.596932	3.391233	2.692658	0.596932	-0.45093	-0.45093	0.596932	-0.87008	0.94622	-0.10164	3.74052	-0.10164	0.596932
0.961407	-0.73011	0.669767	0.961407	-0.26348	0.057322	1.544688	0.961407	1.544688	-0.4093	-2.40558	0.173978	0.378126
0.08471	0.446007	0.103725	-0.20052	-0.7225	0.826319	-0.7225	2.613787	0.008647	-0.0484	0.769272	-0.7225	-0.7225
0.294707	0.294707	-1.16264	2.399759	0.13278	0.456634	-0.83878	0.294707	-0.67686	-0.51493	-0.19107	-0.67686	-0.51493
-0.49728	-0.49728	-0.49728	-0.49728	-0.49728	-0.49728	-0.49728	-0.49728	-0.49728	0.386311	-0.49728	0.28065	-0.49728
1.073365	-0.85506	0.527069	-0.97771	-0.29238	-0.29238	-0.18312	-0.86053	-0.97771	-0.12849	-0.56553	0.090031	1.783551
-0.90686	-0.09569	-0.63647	0.580296	0.039511	-0.50127	1.52667	-0.09569	-0.23088	-0.77167	0.039511	-0.63647	-0.23088
-2.03184	-0.37994	1.271966	0.170697	-0.01285	1.088421	2.740325	-0.01285	-0.74703	0.354242	-0.56348	-1.11412	-0.74703
2.433687	3.348571	-0.44282	-0.44282	-0.44282	1.734069	1.464986	2.810404	1.895519	-0.44282	3.025671	2.003153	-0.44282
0.442544	1.267456	1.006958	-1.00974	-1.00974	-1.00974	0.138629	-1.00974	0.312295	0.225462	1.050374	-1.00974	0.703043
0.400966	0.613381	-0.58384	-0.58384	-0.58384	-0.58384	-0.58384	-0.58384	-0.58384	0.188551	-0.58384	1.197522	-0.58384
0.201499	-0.22464	-0.70759	0.769682	0.059453	0.059453	0.627636	0.201499	-0.4093	-1.58046	-0.08259	1.764001	-0.52293
-0.46485	0.696731	-0.46485	-0.46485	-0.46485	0.163256	-0.46485	0.239467	-0.09078	0.010835	-0.46485	0.239467	-0.46485
-0.70256	0.128835	-0.52182	-0.91221	-0.23264	1.574732	-0.98089	-1.00981	-0.88691	-0.52182	-0.41338	-0.1242	-0.48567
0.982551	-0.32298	-0.37133	0.112199	-0.32298	-1.09662	0.450669	1.46608	-0.46804	0.160552	1.175962	0.595728	-0.80651
-0.29645	1.815833	-0.24789	-0.5878	1.573042	0.723274	0.28625	-0.5878	-0.51496	-0.4664	0.01918	-0.05366	1.08746

[illegible]

control 223	control 224	control 225	control 226	control 227	control 228	control 229	control 230	control 231	control 232	control 233	control 234	control 235	control 236	control 237	control 238	control 239
0.515379	0.976015	0.630538	-0.81413	0.515379	-0.81413	0.745697	-0.81413	0.976015	1.091173	-0.81413	0.860856	-0.81413	0.285061	0.860856	-0.81413	-0.81413
-0.50893	-0.50893	-0.46124	-0.31814	-0.65203	-1.2244	-0.12735	-1.129	-1.98756	-1.70137	-0.50893	-0.65203	-0.65203	-0.36584	-1.46289	-0.03196	-1.74907
2.093388	2.811138	-0.4295	-0.4295	-0.4295	2.811138	-0.4295	-0.4295	-0.4295	-0.4295	-0.4295	-0.4295	-0.4295	2.332638	-0.4295	2.571888	-0.4295
0.172221	-0.50316	-0.50316	-0.44176	-0.62595	-0.99434	0.172221	-0.93294	-2.0995	-1.42412	0.7862	-0.44176	0.172221	0.172221	-1.48552	0.172221	-1.9767
-0.32519	1.065349	-0.09343	-1.21706	0.37008	0.138324	0.215576	0.447332	-0.09343	0.061072	-0.24794	-1.21706	0.061072	-1.21706	-0.09343	0.138324	0.447332
-0.49527	0.171939	-0.62871	-0.49527	4.518318	-0.62871	-0.05682	-0.72403	-0.45714	0.30538	0.629452	-0.51433	-0.15213	-0.11401	-0.13307	-0.53339	0.076624
-0.67253	0.26284	-0.29838	-1.23376	-0.11131	-0.67253	-1.04668	-0.67253	-0.67253	1.572365	-0.67253	-0.48546	0.075765	0.075765	-0.67253	1.01114	-1.23376
-0.32643	-0.32643	-0.32643	-0.32643	1.306666	-0.32643	-0.32643	-0.32643	-0.32643	-0.32643	-0.32643	-0.32643	-0.32643	-0.32643	-0.32643	-0.32643	-0.32643
-0.48062	-0.48062	-0.48062	-0.48062	-0.48062	-0.48062	-0.48062	-0.48062	-0.48062	-0.48062	-0.48062	-0.48062	-0.48062	-0.48062	-0.48062	-0.48062	-0.48062
-0.10164	-0.80022	-1.04472	-0.45093	-0.80022	-0.97486	-0.45093	0.596932	-0.80022	-0.10164	0.247645	-0.905	-0.87008	-0.80022	0.596932	-0.45093	-0.45093
0.961407	-0.61345	-2.40558	-2.40558	-2.40558	0.669767	1.836328	-0.90509	-0.84676	-0.05933	0.144814	0.669767	-1.10924	0.669767	-0.23432	0.11565	-0.49679
0.598132	0.103725	-0.14348	-0.02938	-0.7225	-0.7225	2.613787	-0.7225	-0.7225	0.198804	-0.7225	0.122741	-0.7225	-0.21954	0.008647	-0.7225	-0.7225
-0.51493	-1.00071	-0.353	0.942415	1.104342	-0.19107	-1.16264	2.075905	-0.353	1.913978	0.780488	-1.32456	-0.19107	-0.19107	-1.00071	0.294707	-0.51493
-0.49728	-0.49728	0.624049	-0.49728	-0.49728	0.967449	-0.49728	1.125941	-0.49728	-0.49728	-0.49728	-0.49728	-0.49728	0.386311	-0.49728	1.07311	-0.49728
-0.23775	0.144661	-0.34701	-0.85506	-0.97771	0.30855	1.018736	-0.49451	-0.34701	-0.97771	-0.34701	0.035401	-0.97771	-0.23775	0.745587	-0.97771	-0.97771
-0.09569	1.121081	0.174707	-0.63647	-3.41408	0.174707	-0.36608	0.309904	1.797063	-3.41408	0.039511	-0.09569	-0.09569	-0.77167	0.4451	1.121081	-0.63647
-0.19639	0.354242	-0.56348	0.170697	0.904876	0.354242	-0.37994	3.107415	-0.19639	-0.93057	0.537787	-0.01285	0.170697	-0.19639	-0.19639	1.271966	2.189691
-0.44282	-0.44282	-0.44282	-0.44282	-0.44282	-0.44282	1.357352	-0.44282	-0.44282	-0.44282	1.787886	-0.44282	-0.44282	-0.44282	-0.44282	-0.44282	-0.44282
-1.00974	-1.00974	-1.00974	-1.00974	-1.00974	-1.00974	-1.00974	-1.00974	-1.00974	1.180623	-1.00974	-1.00974	-1.00974	0.355711	0.225462	-1.00974	0.48596
0.719589	-0.58384	-0.58384	-0.58384	0.560277	1.250626	-0.58384	-0.58384	0.135448	-0.58384	-0.58384	-0.58384	0.294759	0.45407	0.241655	-0.58384	-0.58384
1.337864	0.48559	0.769682	0.48559	0.343545	-0.08259	0.201499	0.627636	-0.43771	-1.58046	-0.25305	-1.58046	0.911727	1.764001	-0.22464	0.343545	-0.59396
0.544309	-0.46485	0.061642	-0.46485	-0.46485	-0.46485	-0.46485	-0.46485	0.112449	-0.46485	-0.46485	-0.46485	-0.46485	-0.46485	-0.46485	0.010835	-0.46485

control 252	control 253	control 254	case1 [UIP(+)RA, case101-146] Mean	SD	case2 [NSIP(+)RA, case201-254] Mean	SD	control1[CLD(-)RA, control101-146] Mean	SD	control2[CLD(-)RA, control201-254] Mean	SD	case1+2 [ILD(+)RA] Mean	SD	control1+2[CLD(-)RA] Mean	SD	case1+2 vs. control1+2 P	case1 vs. control1 P	case2 vs. control2 P	meta-analysis of (case1 vs. control1) and (case2 vs. control2) P	meta-analysis of (case1 vs. control1) and (case2 vs. control2) FDR Q
-0.81413	1.091173	1.091173	-0.56525	0.625399	-0.58421	0.618772	0.661029	1.061676	0.529934	0.89962	-0.57549	0.618748	0.589523	0.973687	3.85E-19	3.92E-09	2.07E-11	9.1E-14	1.87E-11
-0.27045	0.397321	0.731204	0.521749	0.887652	0.378772	1.074547	-0.60009	0.74989	-0.28899	0.791676	0.444542	0.990668	-0.4304	0.784649	3.77E-11	2.17E-09	0.000373	1.17E-08	1.2E-06
-0.4295	-0.4295	-0.4295	-0.4295	2.81E-16	-0.4295	5.04E-16	0.325917	1.214181	0.531729	1.342022	-0.4295	1E-15	0.438178	1.283174	1.97E-10	7.37E-05	7.44E-07	1.04E-07	7.09E-06
0.172221	-0.62595	0.7862	0.419147	0.92686	0.32799	1.04371	-0.60412	0.816652	-0.15751	0.867829	0.369922	0.9878	-0.36051	0.869883	6.45E-08	1.44E-07	0.009854	2.04E-06	0.000104
-1.21706	0.138324	0.524584	-0.2712	0.940543	-0.36822	0.891176	0.552524	1.117215	0.140541	0.831708	-0.32359	0.910844	0.327806	0.988442	2.78E-06	0.000287	0.002744	0.000181	0.007406
0.191002	-0.28557	-0.05682	-0.35195	0.804087	-0.24802	0.818468	0.433931	1.001768	0.2083	1.140905	-0.29583	0.809461	0.31086	1.080407	2.01E-05	0.000172	0.018702	0.000562	0.0192
-0.48546	1.946515	-0.11131	0.177437	0.944646	0.391021	1.11822	-0.41063	0.769678	-0.19445	0.942525	0.292772	1.042342	-0.29272	0.8707	2.46E-05	0.001506	0.004007	0.000772	0.022596
-0.32643	-0.32643	-0.32643	0.348786	1.497926	0.198404	1.174751	-0.28105	0.304424	-0.25527	0.333893	0.26758	1.328301	-0.26699	0.319481	0.000123	0.00625	0.007423	0.003967	0.101657
2.364651	2.364651	-0.48062	0.361198	1.251115	0.168651	1.082847	-0.18533	0.76635	-0.313	0.70002	0.257223	1.161262	-0.25497	0.729918	0.000227	0.011995	0.00711	0.00592	0.13484
0.596932	-0.80022	-1.00979	0.055537	0.915662	0.422935	1.109171	-0.26309	0.790708	-0.24265	0.992977	0.253932	1.036147	-0.25194	0.902254	0.000275	0.072497	0.001381	0.006524	0.13484
-0.84676	0.669767	-2.40558	0.224825	1.041571	0.265548	0.732987	-0.22706	0.855115	-0.2233	1.170384	0.246815	0.883837	-0.22501	1.033981	0.000412	0.015459	0.010614	0.008871	0.165319
2.803944	-0.16249	-0.7225	0.380981	1.233339	0.111314	1.102087	-0.26782	0.656064	-0.19929	0.800907	0.235361	1.16615	-0.23044	0.735754	0.000769	0.001917	0.096808	0.008871	0.165319
-0.51493	-0.19107	1.104342	0.143341	1.119993	0.291709	1.12955	-0.31702	0.782605	-0.1431	0.832014	0.223459	1.121939	-0.22215	0.810519	0.001428	0.023913	0.024762	0.016459	0.224317
