# Supplementary material for: Serum Metabolomic Profiling in Rheumatoid Arthritis Patients With Interstitial Lung Disease: A Case–Control Study
Source: Front Med (Lausanne). 2020 Dec 17;7:599794. doi: 10.3389/fmed.2020.599794 (PMC7773768; doi:10.3389/fmed.2020.599794)
Supplement: Supplementary file 3 [file Table_3.pdf]

Supplementary Table S3. Metabolites with higher VIP scores for component 1 of PLS-DA analysis in comparing the RA patients with ILD or without CLD.

	VIP score
Decanoic acid	4.8800
Glycerol	3.7696
Morpholine	3.6421
Dyphylline	3.1369
Octanoic acid	2.7440
Fumaric acid monomethyl ester	2.5086
N-Acetylgalactosamine-1<br>N-Acetylmannosamine-1<br>N-Acetylglucosamine-1	2.4827

VIP: variable importance in projection, PLS-DA: Partial Least Squares-Discriminant Analysis, RA: rheumatoid arthritis, ILD: interstitial lung disease, CLD: chronic lung disease.
