# Supplementary material for: Serum Metabolomic Profiling in Rheumatoid Arthritis Patients With Interstitial Lung Disease: A Case–Control Study
Source: Front Med (Lausanne). 2020 Dec 17;7:599794. doi: 10.3389/fmed.2020.599794 (PMC7773768; doi:10.3389/fmed.2020.599794)
Supplement: Supplementary file 4 [file Image_1.pdf]

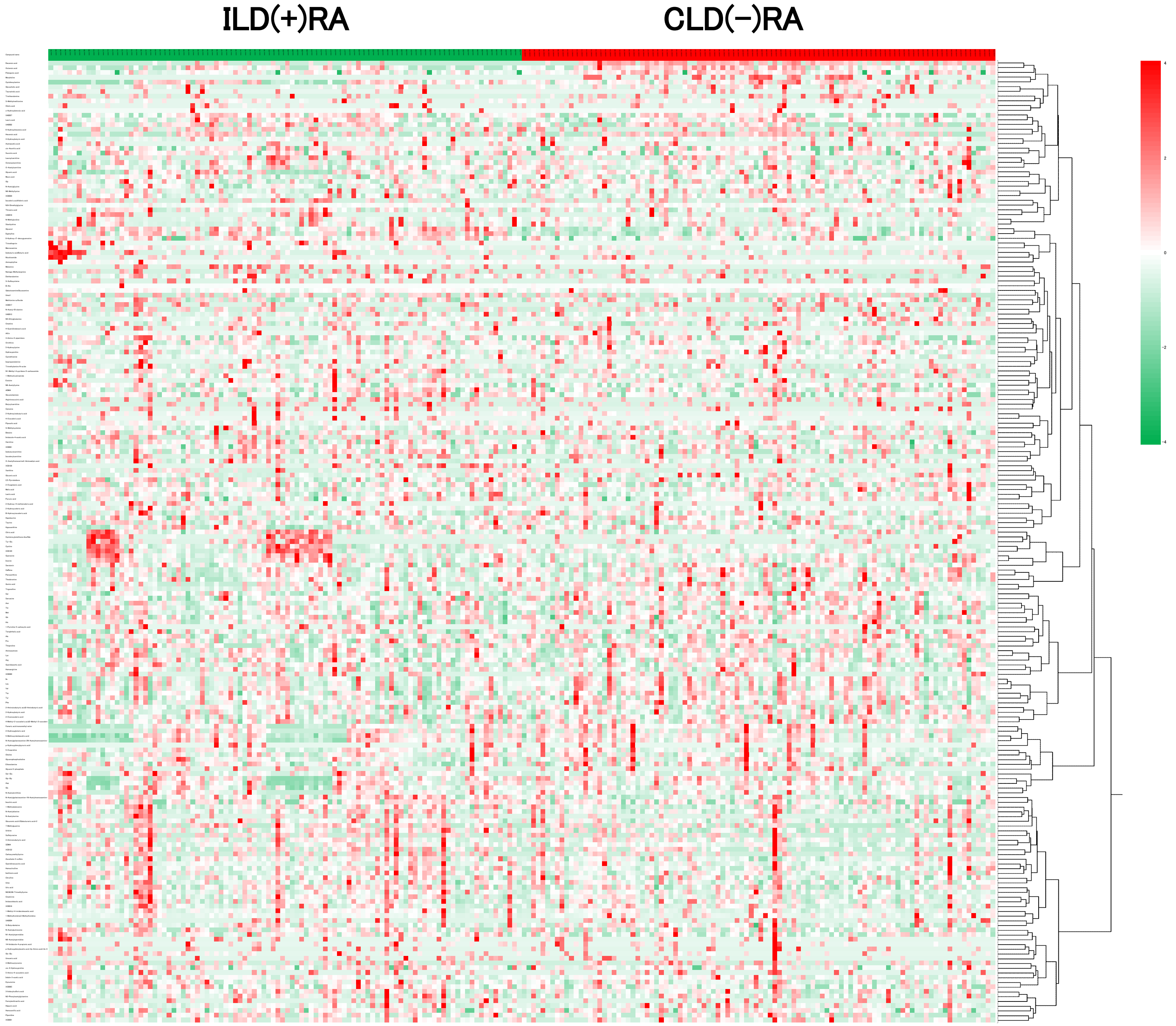

Supplementary Figure S1. Hierarchical clustering analysis of serum metabolites from the RA patients with ILD or without CLD. Each column and raw indicate a sample from an RA patient and a metabolite, respectively. The normalized levels of the metabolites were listed in Supplementary Table S2. RA: rheumatoid arthritis, ILD: interstitial lung disease, CLD: chronic lung disease, ILD(+)RA: RA patients with ILD, CLD(−)RA: RA patients without CLD.
