# Supplementary material for: Serum Metabolomic Profiling in Rheumatoid Arthritis Patients With Interstitial Lung Disease: A Case–Control Study
Source: Front Med (Lausanne). 2020 Dec 17;7:599794. doi: 10.3389/fmed.2020.599794 (PMC7773768; doi:10.3389/fmed.2020.599794)
Supplement: Supplementary file 5 [file Image_2.pdf]

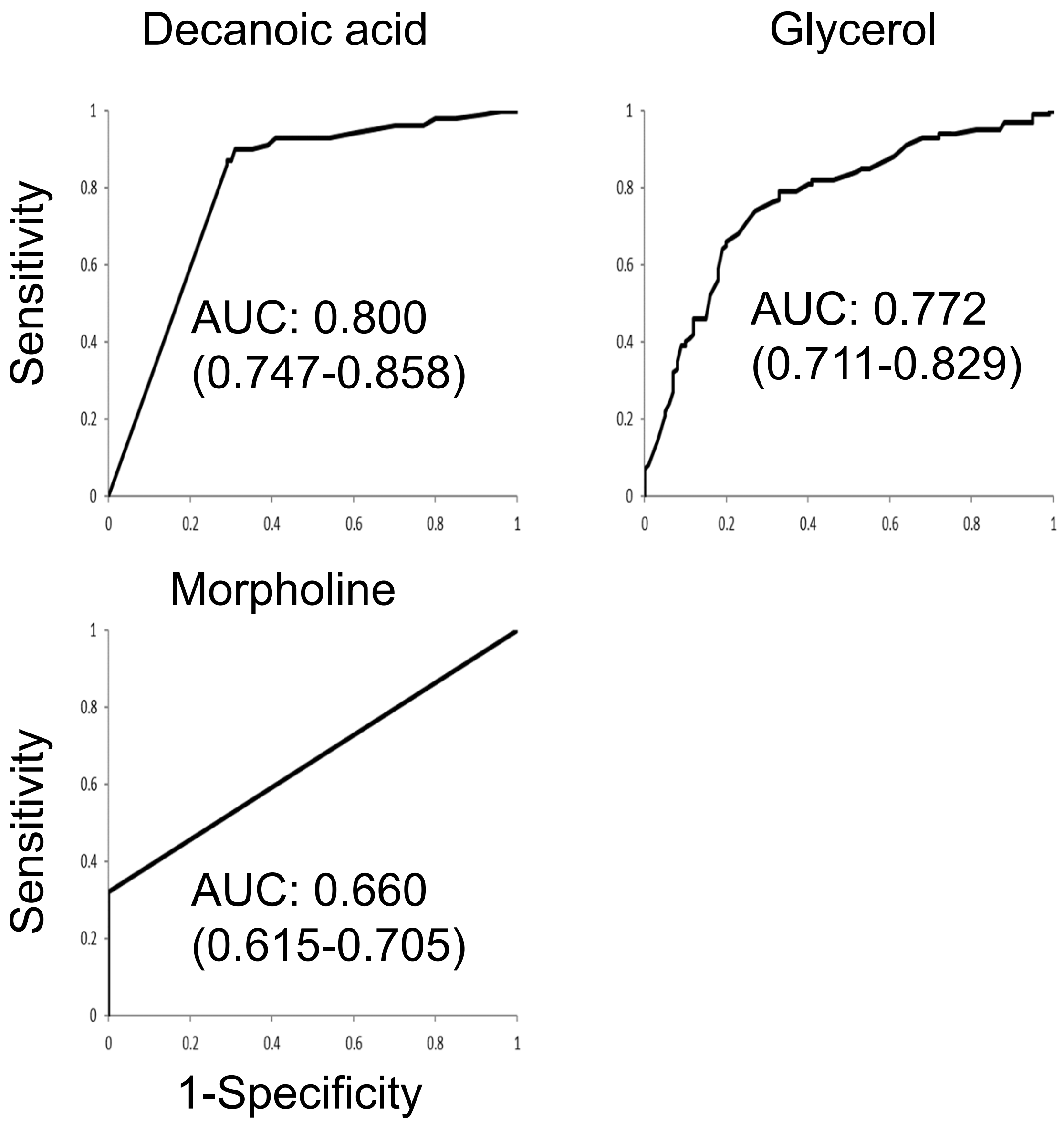

Supplementary Figure S2. ROC curves of three metabolites with higher VIP scores comparing RA patients with ILD or without CLD. ILD: interstitial lung disease, AUC: area under the curve, RA: rheumatoid arthritis, ILD: interstitial lung disease, ROC: receiver operating characteristic, VIP: variable importance in projection.
