# Supplementary material for: Serum Metabolomic Profiling in Rheumatoid Arthritis Patients With Interstitial Lung Disease: A Case–Control Study
Source: Front Med (Lausanne). 2020 Dec 17;7:599794. doi: 10.3389/fmed.2020.599794 (PMC7773768; doi:10.3389/fmed.2020.599794)
Supplement: Supplementary file 6 [file Image_3.pdf]

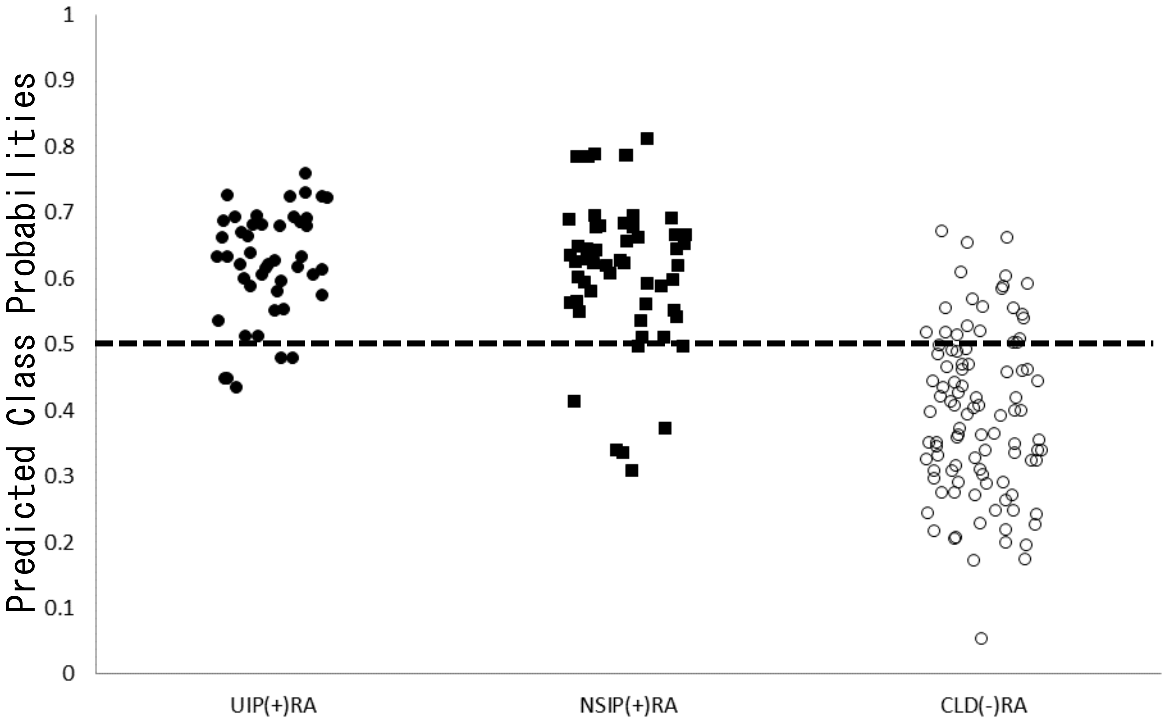

Supplementary Figure S3. Plot of the predicted class probabilities for samples from UIP(+)RA, NSIP(+)RA, and CLD(-)RA using the PLS-DA model created with the three metabolites. The cutoff value was 0.5 due to balanced subsampling. UIP(+)RA: rheumatoid arthritis patients with usual interstitial pneumonia, NSIP(+)RA: rheumatoid arthritis patients with non-specific interstitial pneumonia, CLD(-)RA: rheumatoid arthritis patients without chronic lung diseases, PLS-DA: partial least squares-discriminant analysis.
